# Palmitoylation regulates myelination by modulating the ZDHHC3-Cadm4 axis in the central nervous system

**DOI:** 10.1038/s41392-024-01971-5

**Published:** 2024-09-26

**Authors:** Yanli Chang, Jiangli Zhu, Xiaopeng Li, Yi Deng, Birou Lai, Yidan Ma, Jia Tong, Huicong Liu, Juanjuan Li, Chenyu Yang, Qiao Chen, Chengbiao Lu, Yinming Liang, Shiqian Qi, Xiaoning Wang, Eryan Kong

**Affiliations:** 1 The Third Affiliated Hospital of Xinxiang Medical University, Xinxiang, China; 2 Institute of Psychiatry and Neuroscience, Xinxiang Key Laboratory of Protein Palmitoylation and Major Human Diseases, Xinxiang Medical University, Xinxiang, China; 3 Department of Urology, State Key Laboratory of Biotherapy and Cancer Center, West China Hospital, Sichuan University and National Collaborative Innovation Center, Chengdu, China; 4 Center of Cryo-Electron Microscopy, Zhejiang University, Hangzhou, China; 5 Department of Nutrition, Third Medical Center of PLA General Hospital, Beijing, China; 6 School of Life Sciences, Fudan University, Shanghai, China

**Keywords:** Neurodevelopmental disorders, Molecular medicine

## Abstract

The downregulation of Cadm4 (Cell adhesion molecular 4) is a prominent feature in demyelination diseases, yet, the underlying molecular mechanism remains elusive. Here, we reveal that Cadm4 undergoes specific palmitoylation at cysteine-347 (C347), which is crucial for its stable localization on the plasma membrane (PM). Mutation of C347 to alanine (C347A), blocking palmitoylation, causes Cadm4 internalization from the PM and subsequent degradation. In vivo experiments introducing the C347A mutation (Cadm4-KI) lead to severe myelin abnormalities in the central nervous system (CNS), characterized by loss, demyelination, and hypermyelination. We further identify ZDHHC3 (Zinc finger DHHC-type palmitoyltransferase 3) as the enzyme responsible for catalyzing Cadm4 palmitoylation. Depletion of ZDHHC3 reduces Cadm4 palmitoylation and diminishes its PM localization. Remarkably, genetic deletion of ZDHHC3 results in decreased Cadm4 palmitoylation and defects in CNS myelination, phenocopying the Cadm4-KI mouse model. Consequently, altered Cadm4 palmitoylation impairs neuronal transmission and cognitive behaviors in both Cadm4-KI and ZDHHC3 knockout mice. Importantly, attenuated ZDHHC3-Cadm4 signaling significantly influences neuroinflammation in diverse demyelination diseases. Mechanistically, we demonstrate the predominant expression of Cadm4 in the oligodendrocyte lineage and its potential role in modulating cell differentiation via the WNT-β-Catenin pathway. Together, our findings propose that dysregulated ZDHHC3-Cadm4 signaling contributes to myelin abnormalities, suggesting a common pathological mechanism underlying demyelination diseases associated with neuroinflammation.

## Introduction

Myelination is a critical process within the nervous system that ensures rapid and efficient neural communication, facilitating swift and coordinated responses in living organisms.^1,2^ Central to this process is the myelin sheath, a specialized insulating layer that encases the axons of neurons.^3^ This sheath is indispensable for the saltatory conduction of action potentials, a mechanism that significantly enhances the speed and efficiency of electrical signal transmission along nerve cells.^3,4^ The myelin sheath is formed through the complex wrapping of glial cell membranes around the axonal membrane, resulting in an insulating layer that enables electrical signals to jump between the nodes of Ranvier.^2^ This leapfrogging of the action potential substantially accelerates conduction velocity compared to continuous conduction along the entire axonal length.^2,5^ The formation and maintenance of the myelin sheath involve intricate cellular and molecular interactions. In the central nervous system, oligodendrocytes are the glial cells responsible for myelinating axons, while Schwann cells perform this function in the peripheral nervous system (PNS).^1,6^ These glial cells extend their membranes around the axonal shaft, creating multiple concentric layers that constitute the myelin sheath. This wrapping process is regulated by a variety of cell surface proteins and adhesion molecules, including cell adhesion molecules (Cadms),^7,8^ which are crucial for ensuring proper myelin formation. Cadms facilitate interactions between glial cells and axons, promoting the alignment and stabilization of the myelin sheath.^8,9^

Cadms, also known as synaptic cell adhesion molecules (SynCAM) or Nectin-like adhesion molecules (Necl), are characterized by their unique structural features.^7,10^ Each Cadm molecule possesses a single transmembrane domain, extracellular immunoglobulin (Ig)-like domains, and a short cytoplasmic tail. The extracellular Ig-like domains are essential for mediating heterophilic interactions between Cadms on adjacent cells, which are necessary for correct myelin sheath alignment and maintenance.^7,8,10^ The intracellular tails of Cadms contain binding motifs for various proteins, such as 4.1 G and PDZ sequences, which connect Cadms to the cytoskeleton and intracellular signaling pathways.^11,12^ The Cadm family consists of four members: Cadm1, Cadm2, Cadm3, and Cadm4. Of these, Cadm1, Cadm2, and Cadm3 are expressed on both glial cells and axons, while Cadm4 is predominantly found on glial cells.^7,9^ Interactions between Cadm2/3 and Cadm4 are particularly significant for myelination, as they facilitate proper alignment and stabilization of the myelin sheath. It has been observed that the expression levels of Cadms must be tightly regulated to ensure efficient myelination. Both excessive and insufficient expression of Cadm4 and Cadm3 can disrupt the myelination process, leading to impaired nerve function and contributing to various neurological disorders.^7,8^ One notable condition associated with disrupted Cadm4 function is Charcot-Marie-Tooth (CMT) neuropathy,^13^ a hereditary demyelinating disease characterized by progressive muscle weakness and sensory loss. Research has demonstrated that decreased levels of Cadm4 are a common feature in CMT neuropathy,^13^ indicating the crucial role of Cadm4 in maintaining normal nerve function. Despite these associations, the precise molecular mechanisms governing Cadm4 regulation and its role in demyelinating diseases remain inadequately understood.

Recent advancements in proteomics have provided new insights into the post-translational modifications of endogenous proteins,^14^ particularly the role of palmitoylation.^15,16^ This reversible modification involves the addition of palmitate, a 16-carbon saturated fatty acid, to cysteine residues, significantly affecting several characteristics of the palmitoylated protein, such as increased hydrophobicity, altered membrane localization, modified protein-protein interactions, and changes in downstream signaling pathways.^17–19^ This dynamic process is catalyzed by palmitoyltransferases, also known as the ZDHHC family (ZDHHC 1 to 9 and 11 to 25; there is no known isoform 10), which add palmitate, and by enzymes like APT1/2, PPT1/2, and ABHD17a, which remove it.^20,21^ Our research employed palm-proteomics to identify palmitoylated proteins in the CNS,^15,22,23^ leading to the discovery that Cadm4 is subject to palmitoylation. This finding prompted further investigation into the role of palmitoylation in Cadm4 function and its implications for myelination. Moreover, we identified ZDHHC3, a member of the ZDHHC family, as the key enzyme responsible for mediating Cadm4 palmitoylation. Notably, this modification is essential for maintaining the stable presence of Cadm4 on the PM, which is crucial for proper CNS myelination.

To elucidate the significance of the ZDHHC3-Cadm4 palmitoylation axis, we employed a multidisciplinary approach incorporating genetically engineered mouse models, biochemical assays, point mutations, and electron microscopy. Our findings revealed that the ZDHHC3-Cadm4 axis plays a critical, previously unrecognized role in CNS myelination. Unexpectedly, alterations in Cadm4 palmitoylation are closely linked to neuroinflammation in various demyelination mouse models. This neuroinflammation correlates with impaired cognitive functions and suggests that disruptions in the ZDHHC3-Cadm4 palmitoylation axis may contribute to the pathogenesis of several demyelinating diseases. These results underscore the potential of targeting the ZDHHC3-Cadm4 palmitoylation axis as a therapeutic strategy for treating demyelinating conditions associated with neuroinflammation. By advancing our understanding of the molecular mechanisms governing Cadm4 regulation and its role in myelination, our research aims to provide new insights and approaches for addressing these challenging neurological disorders. Ultimately, the goal is to develop novel interventions that can improve the treatment and management of diseases characterized by demyelination and neuroinflammation.

## Results

### Cadm4 is specifically palmitoylated at cysteine-347

**Fig. 1:**
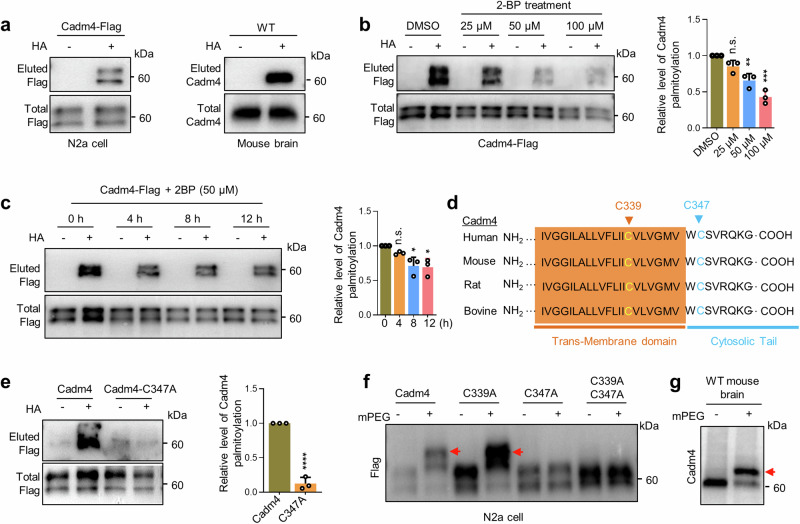
Cadm4 is specifically palmitoylated at cysteine-347. **a** Cadm4 expressed in N2a cells or from mouse brain was analyzed for protein palmitoylation by Acyl-RAC assay, and quantified. HA+, with hydroxylamine, HA−, without hydroxylamine. **b**, **c** N2a cells expressing Cadm4-Flag was incubated with 2-BP for various dose and time, and were evaluated for the level of palm-Cadm4, and quantified (*n* = 3 biological replicates, one-way ANOVA; *p ≤ 0.05, **p ≤ 0.01, ****p ≤ 0.0001, n.s. for not significant). **d** Protein sequences of Cadm4 from various species were aligned for analyzing cysteine conservation. **e** Cadm4 or its mutants (C347A and C339A/C347A) were expressed in N2a cells and subjected for Acyl-RAC assay, and quantified (two-tailed *t*-test, *n* = 3, ****p ≤ 0.0001). Cadm4 and its mutants (C347A and C339A/C347A) expressed in N2a cells (**f**) or endogenously expressed Cadm4 (**g**) were processed with mPEG-labeling assay. The mPEG labeling causes the band shift, red arrows pointed. Data are represented as mean ± SEM

The initial attempt to identify palmitoylated proteins in CNS by palm-proteomics,^15^ we spotted Cadm4, implying that Cadm4 is palmitoylated (Supplementary Fig. 1a). To verify if Cadm4 is indeed modified with protein palmitoylation, Cadm4 expressed either ectopically in N2a, a neuroblastoma cell line, HEK-293T or expressed endogenously in WT mouse brain was evaluated by Acyl-RAC assay.^15,24^ The results showed that Cadm4 is readily palmitoylated both in vitro and in vivo (Fig. 1a and Supplementary Fig. 1b). For confirmation, 2-BP (an inhibitor of protein palmitoylation) was used to incubate with cells expressing Cadm4-Flag, the experiments showed that 2-BP could effectively reduce the level of palmitoylated-Cadm4 (palm-Cadm4) in a time and dose dependent manner (Fig. 1b, c). Curiously, we noticed that multiple bands of Cadm4 appear in WB when Cadm4-Flag is expressed either in N2a or HEK-293T cells (Fig. 1a, b and Supplementary Fig. 1b), interestingly, PNG treatment (an inhibitor of protein glycosylation) could flatten these bands into a single band (Supplementary Fig. 1c).

To identify the cysteine residue that is palmitoylated, we analyzed the protein sequence of Cadm4 by specifically looking at its transmembrane (TM) domain, considering that palmitoylated proteins often associate with hydrophobic membranes.^18,21^ The analysis showed that cysteine-339 (C339) and C347 are close to the boundary of the TM domain and conserved in different species (Fig. 1d). Accordingly, point mutations were introduced into Cadm4. The Acyl-RAC assay showed that while Cadm4 is palmitoylated, the single mutation in C347 (C347A) almost entirely blocks its palmitoylation (Fig. 1e and Supplementary Fig. 1d), suggesting that C347 is the major site of palmitoylation in Cadm4. To confirm this finding, the mPEG labeling assay, where palmitate is substituted by mPEG,^20^ was performed to show that the C347A mutation could fully block the labeling of mPEG-Cadm4 (displayed by no migrated band), yet the WT Cadm4 or Cadm4-C339A can be labeled by mPEG (single migrated band, Fig. 1f and Supplementary Fig. 1e, f is a positive control). The single migrated mPEG-Cadm4 band (arrow pointed, Fig. 1f) suggests that Cadm4 is likely modified with single site of palmitoylation. Furthermore, the mPEG assay demonstrated that endogenously expressed Cadm4 is also palmitoylated at single site since only one migrated mPEG-Cadm4 band was visualized (arrow pointed, Fig. 1g). The additional trial using mass spectrometry failed to capture the peptide covering C347 in Cadm4, leading to an unsuccessful outcome (Supplementary Fig. 1h, i). Together, these data verified that Cadm4 is palmitoylated at C347.

### Palmitoylation regulates Cadm4 for dynamic PM localization

**Fig. 2:**
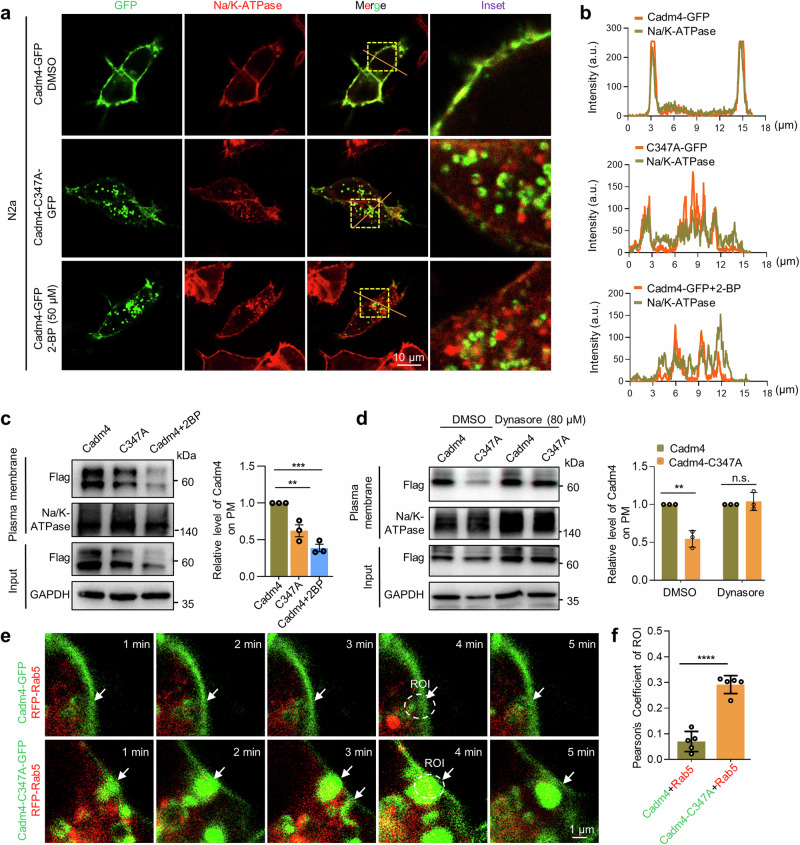
Palmitoylation regulates Cadm4 for dynamic PM localization. **a** N2a cells expressing either Cadm4 or Cadm4-C347A were fixed for immunofluorescence analysis, Na/K-ATPase is a marker for PM. **b** Intensity of the red and green fluorescence was profiled along the slash. **c** N2a cells expressing Cadm4 or Cadm4-C347A were treated with DMSO or 2-BP for preparing plasma membrane fractions, which were evaluated by WB and quantified (one-way ANOVA followed by Bonferroni post hoc test, *n* = 3, **p = 0.009; ***p = 0.001). **d** N2a cells expressing Cadm4 or Cadm4-C347A were treated with DMSO or Dynasore for preparing plasma membrane fractions, which were evaluated by WB and quantified (two-tailed *t*-test, *n* = 3, **p ≤ 0.01). Live-cell imaging of N2a cells expressing Cadm4-GFP/Cadm4-C3471-GFP with RFP-Rab5 (**e**), for colocalization analysis (**f**). Data are represented as mean ± SEM

To test if palmitoylation is involved in regulating Cadm4 for PM localization, Cadm4-GFP and Cadm4-C347A-GFP were expressed in N2a cells. The imaging data showed that as Cadm4 resides mainly on PM, most of Cadm4-C347A distributes within cytosol as vesicle-like structures or puncta (Fig. 2a, b), suggesting that blocking palmitoylation weakens PM localization of Cadm4. Similarly, the treatment of 2-BP with N2a cells expressing Cadm4-GFP also induces cytosolic puncta (Fig. 2a, b). In consistent, biochemical analysis of plasma membrane fraction (Supplementary Fig. 1g) verified that attenuated level of palm-Cadm4, induced either by C347A or 2-BP treatment, significantly weakens PM localization of Cadm4 as compared to that of WT Cadm4 (Fig. 2c), yet, interestingly, the application of Dynasore (inhibitor of dynamin-dependent and clathrin-mediated internalization) almost entirely recovers the PM localization of Cadm4-C347A (Fig. 2d), indicating that the above-mentioned cytosolic puncta is likely internalized vesicles from PM. Indeed, by the approach of live-cell imaging, it was presented that Cadm4-GFP is relatively stable on PM, while Cadm4-C347A-GFP is prone to be internalized, as illustrated by the colocalization of Cadm4-C347A/Rab5 (earlier endosome) at cell edge (Fig. 2e, f). Consistently, the incubation of Dynasore in live-cell inhibits the internalization of Cadm4-C347A and significantly recovers its fluorescence signal on PM (Supplementary Fig. 2a, b). Hereby, these data supported that palmitoylation is vital for the stable PM localization of Cadm4, attenuated level of palm-Cadm4 is prone to be internalized as cytosolic puncta.

### Internalized depalmitoylated-Cadm4 is targeted for protein degradation

**Fig. 3:**
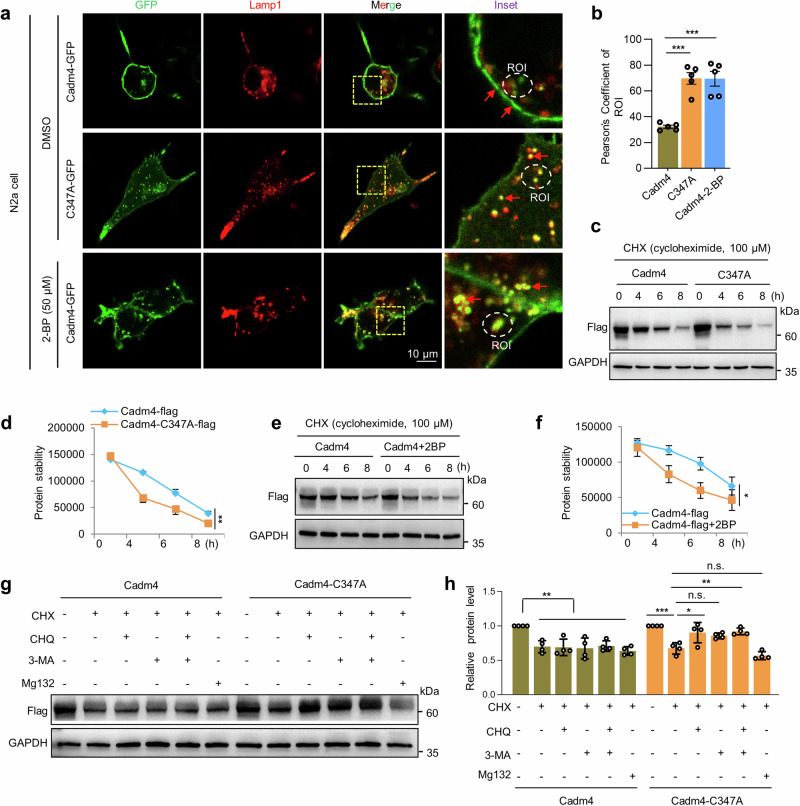
Internalized depalmitoylated Cadm4 is targeted for protein degradation. **a** Cadm4 or Cadm4-C347A was expressed in N2a cells, treated with DMSO or 2-BP (50 µM) and fixed for immunofluorescence analysis, Lamp1 is a marker for lysosome. **b** The colocalization of Cadm4/Lamp1 was quantified (one-way ANOVA followed by Bonferroni post hoc test, *n* = 5, ***p < 0.001). N2a cells expressing Cadm4 or Cadm4-C347A were incubated with CHX (cycloheximide, 100 µM) for various length of time, lysates of which were subjected for WB analysis (**c**) and the levels of Cadm4 were quantified (**d**). **p = 0.007, paired *t*-test, *n* = 3. N2a cells expressing Cadm4 treated with DMSO or 2-BP (50 µM) were incubated with CHX for different periods, analyzed by WB (**e**) and the levels of Cadm4 were quantified (**f**). **p = 0.018, paired *t*-test, *n* = 3. N2a cells expressing Cadm4 or Cadm4-C347A were incubated with or without CHX, CHQ (Chloroquine, 50 µM), 3-MA (Methyladenine, 3 mM) or Mg132 (25 µM), and subjected for WB analysis (**g**), and quantified (**h**). Data are represented as mean ± SEM

To track the fate of internalized cytosolic puncta, RFP-Lamp1 (lysosome) or -Rab7 (late endosome) was coexpressed for colocalization analysis. The imaging data showed that the colocalization of cytosolic puncta with Lamp1 or Rab7 is significantly upregulated when Cadm4-C347A is expressed or upon the treatment of 2-BP as compared to that of the control (Fig. 3a, b and Supplementary Fig. 2c, d), implying that internalized cytosolic puncta is targeted for protein degradation. For further verification, we thought to examine the protein stability of Cadm4, and cycloheximide (CHX) was used to block de novo protein synthesis in N2a cells. As expected, the experiments showed that reducing the level of palm-Cadm4 (C347A mutation or 2-BP treatment) significantly enhances the degradation of Cadm4 as compared to that of the control (Fig. 3c–f). Moreover, to identify the potential pathway that is involved in degrading Cadm4, inhibitors as CHQ (Chloroquine, blocking lysosome degradation) and 3MA (3-Methyladenine, blocking autophagy) were applied. The experiments showed that either 3-MA or CHQ, but not MG132, could markedly inhibit the degradation of Cadm4-C347A (Fig. 3g, h), indicating that autophagy-lysosome pathway is likely engaged for degrading Cadm4-C347A. In brief, these evidences clarified that internalized depalmitoylated-Cadm4 is targeted for degradation via likely autophagy and lysosome pathway.

### Blocking Cadm4 palmitoylation results in severe defects in CNS myelination

**Fig. 4:**
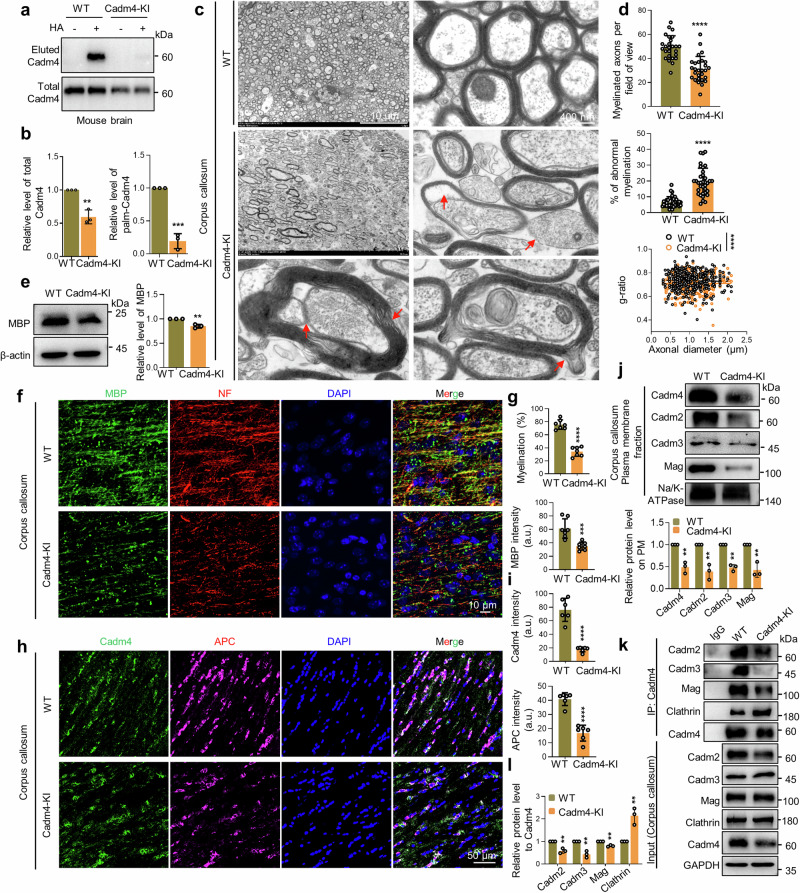
Blocking Cadm4 palmitoylation results in severe defects in CNS myelination. Brain lysates from WT and Cadm4-KI mouse were evaluated for the level of Cadm4 and palm-Cadm4 (**a**), and quantified (**b**, two-tailed *t*-test, *n* = 3, **p ≤ 0.01, ***p ≤ 0.001). **c**, **d** Corpus callosum was isolated from prefixed brains of WT and Cadm4-KI mouse and examined for myelination under electron microscopy. Abnormal myelination is pointed out by red arrows: loss of myelination, demyelination, thickened myelin sheath/hypermyelination, myelination contains multiple axons (**c**); myelinated axons per field of view (****p < 0.0001, two-tailed *t*-test, *n* = 26–28), % of abnormal myelination (****p < 0.0001, two-tailed *t*-test, *n* = 32–35), and g-ratio (****p < 0.0001, two-tailed *t*-test, *n* = 372–415) were quantified from 4–6 mice (**d**). **e** Lysates of corpus collosum from WT and Cadm4-KI mice were evaluated for MBP level by WB, and quantified (two-tailed *t*-test, *n* = 3, **p ≤ 0.01). Mice brains were processed for transparency with X-clarity and stained for MBP, NF (neurofilament) and DAPI for imaging (**f**), % of myelination and MBP fluorescence intensity were quantified (**g**, two-tailed *t*-test, *n* = 6, ***p ≤ 0.001, ****p < 0.0001). Frozen coronal sections of corpus callosum were stained with Cadm4, APC and DAPI for imaging (**h**), the fluorescence intensity of Cadm4 and APC was quantified (**i**, two-tailed *t*-test, *n* = 6, ****p < 0.0001). **j** PM fractions were prepared from corpus callosum of WT and Cadm4-KI mice and analyzed for the levels of Cadms and Mag by WB and quantified (two-tailed *t*-test, *n* = 3, **p ≤ 0.01). Immunoprecipitations were performed with the brain lysates of WT and Cadm4-KI mouse by using Cadm4 antibody, the IP eluent was analyzed for Cadm4 binding proteins (**k**), and quantified (**l**, two-tailed *t*-test, *n* = 3, **p ≤ 0.01). Data are represented as mean ± SEM

As attenuated palm-Cadm4 is targeted for protein degradation and the fact that Cadm4 is important for myelination, one might speculate that blocking Cadm4 palmitoylation in vivo might interfere myelin formation. For verification, a point mutation (C347A) was introduced into Cadm4 to block Cadm4 palmitoylation in C57BL/B6 mouse (hereafter named Cadm4-KI) (Supplementary Fig. 3a, b). Indeed, Cadm4 palmitoylation is largely blocked in Cadm4-KI as compared to that of the WT mouse (Fig. 4a, b), although the Cadm4-KI mouse seems normal at birth.

Initially, the expression profile of Cadm4 is examined, the results showed that Cadm4 is predominantly expressed in oligodendrocytes (Supplementary Fig. 3d) and in a developmental-dependent manner (Supplementary Fig. 3c). For evaluating CNS myelination, corpus callosum of 3-months old mouse were prepared for electron microscopy (Fig. 4c). The results showed that the myelinated axons per field of view is dramatically downregulated (Fig. 4d), while the percentage of abnormal myelination, e.g., demyelination, thickened myelin sheath/hypermyelination, and myelin contains multiple axons is significantly upregulated in Cadm4-KI as compared to that of the WT mouse (Fig. 4d, arrow pointed). Furthermore, the value of g-ratio is significantly reduced in Cadm4-KI as comparing to WT mouse (Fig. 4d), possibly caused by the fact that a proportion of the myelin sheath is markedly thickened in Cadm4-KI mouse.

To briefly explore the cellular basis underlying the abnormal myelination phenotype, the myelin maturation markers, Myelin Basic Protein (MBP) and APC (Adenomatous Polyposis Coli) were used. The results showed that MBP expression (Fig. 4e) or its fluorescence intensity (Fig. 4f, g) is dramatically decreased in Cadm4-KI as compared to that of WT mouse, which may explain the downregulation of myelination in Cadm4-KI mouse (Fig. 4g). Simultaneously, the fluorescence intensity of APC and Cadm4 is also significantly decreased in Cadm4-KI as compared to WT mouse (Fig. 4h, i). At molecular level, the biochemical data showed that the PM localization of Cadm4, as well as other cell adhesion molecules e.g., Cadm2-3 and Mag is significantly decreased in corpus callosum of Cadm4-KI as compared to that of WT control (Fig. 4j), which could result in the dissociation of protein-protein binding between Cadm4 and other cell adhesion molecules, but enhance the binding of Cadm4 with Clathrin (Fig. 4k, l). In total, these results suggest the scenario that blocking Cadm4 palmitoylation reduces its localization and association with Cadm2/3 and Mag on PM, meanwhile enhances its binding with Clathrin for internalization, jointly they cause severe defects in the maturation of oligodendrocytes, and CNS myelination.

### ZDHHC3 mediates Cadm4 palmitoylation and regulates its PM localization and protein stability

To identify the potential enzyme that palmitoylates Cadm4, ZDHHCs were coexpressed with Cadm4 in N2a cells for evaluating palm-Cadm4, the results showed that ZDHHC3 significantly enhances the level of palm-Cadm4 (Supplementary Fig. 4a). For verification, ZDHHC3-KO N2a cell (Using the strategy of disrupting enzyme activity center as in Supplementary Fig. 5; due to the issue of ZDHHC3 antibody, the evidence at protein level cannot be shown) was constructed, the Acyl-RAC assay showed that the deletion of ZDHHC3 significantly diminishes the level of palm-Cadm4 (Supplementary Fig. 4b). Furthermore, immunoprecipitation experiments confirmed that ZDHHC3 interacts with Cadm4 (Supplementary Fig. 4c), and they colocalize nicely at Golgi apparatus, yet interestingly, Cadm4-C347A does not colocalize with ZDHHC3 at all (Supplementary Fig. 4d).

**Fig. 5:**
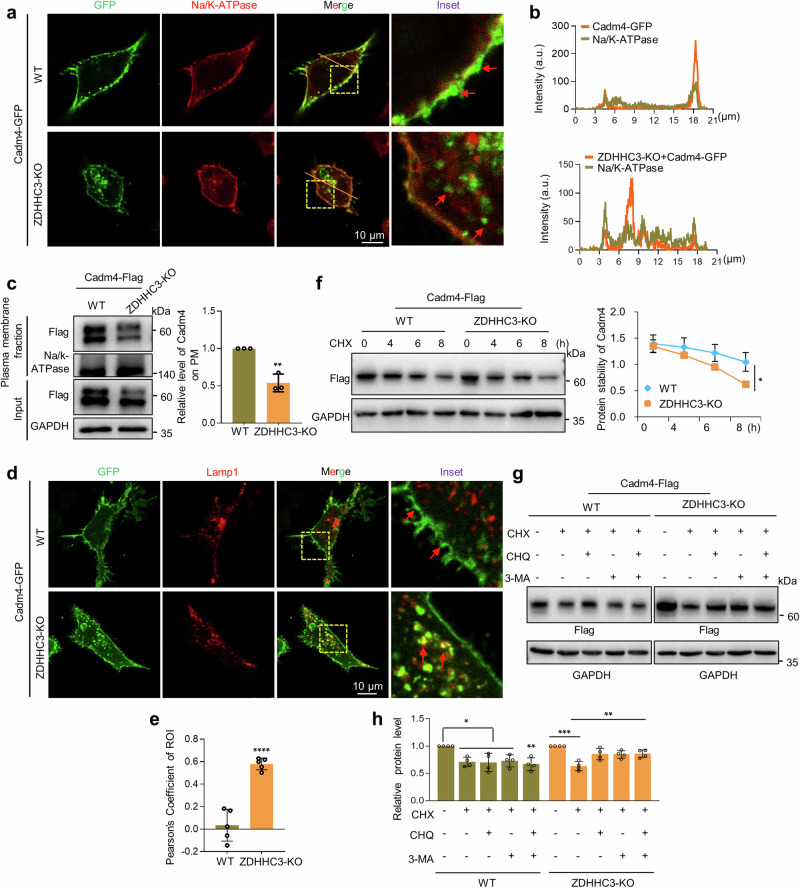
ZDHHC3 mediates Cadm4 palmitoylation and regulates its PM localization and protein stability. Cadm4-GFP was expressed in WT and ZDHHC3-KO cells, and fixed for immunofluorescence imaging (**a**), Na/K-ATPase is a marker for PM, the profile of the intensity of the red and green fluorescence was measured along the slash (**b**). **c** PM fractions were prepared from WT or ZDHHC3-KO cells expressing Cadm4 and subjected for WB analysis, and quantified (two-tailed *t*-test, *n* = 3, **p ≤ 0.01). Cadm4-GFP was expressed in WT and ZDHHC3-KO cells and fixed for immunofluorescence imaging (**d**), Lamp1 is a marker for lysosome; the colocalization of Cadm4/Lamp1 was analyzed (**e**, ****p < 0.0001, two-tailed *t*-test, *n* = 5). **f** WT or ZDHHC3-KO cells expressing Cadm4-Flag were incubated with CHX (100 µM) for different period, subjected for WB analysis and quantified (paired *t*-test, *p < 0.05). WT or ZDHHC3-KO cells expressing Cadm4-Flag were incubated with or without CHX, CHQ (50 µM) or 3-MA (3 mM) and subjected for WB analysis (**g**), and quantified (**h**, one-way ANOVA followed by Bonferroni post hoc test, *n* = 4, *p ≤ 0.05, **p ≤ 0.01, ***p < 0.001). Data are represented as mean ± SEM

Next, we thought to examine if PM localization of Cadm4 is affected in ZDHHC3-KO cell. The imaging figures showed that Cadm4 localizes mainly on PM in WT cell, while a large proportion of Cadm4 relocates into cytosol as puncta in ZDHHC3-KO cell (Fig. 5a, b), part of which is also supported by the biochemical data (Fig. 5c). Not surprisingly, these cytosolic puncta colocalizes with Rab7 (Supplementary Fig. 4e) or lysosome marker Lamp1 (Fig. 5d, e), indicating that they are targeted for degradation. Moreover, Cadm4 protein stability was tested, the CHX-treatment experiments showed that as ZDHHC3 is absent, the degradation of Cadm4 is accelerated (Fig. 5f). Consistently, such accelerated Cadm4 degradation can be inhibited by the treatment of 3-MA or CHQ, suggesting of the involvement of autophagy and lysosome pathway (Fig. 5g, h). Jointly, the above data strengthened the notion that Cadm4 palmitoylation, mediated by ZDHHC3, is vital for its PM localization and protein stability.

### Loss of ZDHHC3 virtually phenocopies the defects of myelination in Cadm4-KI mouse

**Fig. 6:**
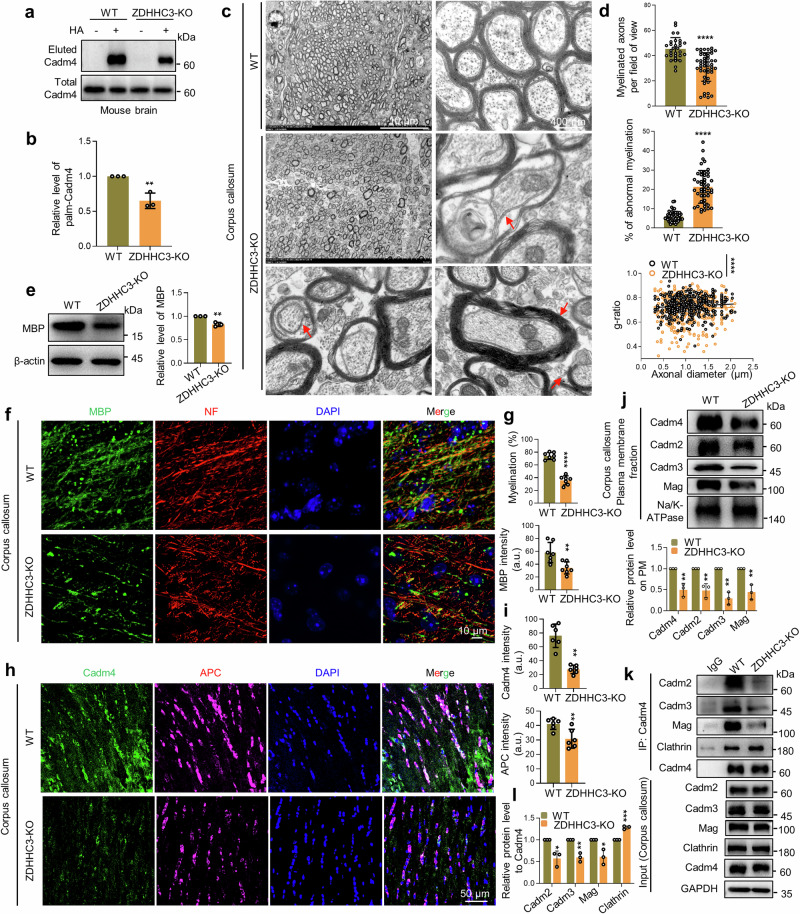
Loss of ZDHHC3 virtually phenocopies the defects of myelination in Cadm4-KI. Brain lysates of WT and ZDHHC3-KO mice were evaluated for the level of palm-Cadm4 by Acyl-RAC assay (**a**), and quantified (**b**, two-tailed *t*-test, *n* = 3, **p ≤ 0.01). **c**, **d** Corpus callosum was isolated from prefixed brains of WT and ZDHHC3-KO mice and examined for myelination under electron microscopy. Abnormal myelination is pointed out by red arrows: loss of myelination, detachment of axon from myelin sheath, thickened myelin sheath/hypermyelination (**c**); myelinated axons per field of view (****p < 0.0001, *t*-test, *n* = 31–49), % of abnormal myelination (****p < 0.0001, *t*-test, *n* = 48–50) and g-ratio (****p < 0.0001, *t*-test, *n* = 414–476) were quantified from 4–6 mouse (**d**). **e** Lysates of corpus collosum from WT and ZDHHC3-KO mice were evaluated for MBP level by WB, and quantified (two-tailed *t*-test, *n* = 3, **p ≤ 0.01). Mice brains were processed for transparency with X-clarity and stained for MBP, NF (neurofilament) and DAPI for imaging (**f**), % of myelination and MBP fluorescence intensity were quantified (**g**, two-tailed *t*-test, *n* = 6, **p ≤ 0.01, ****p < 0.0001). Frozen coronal sections of corpus callosum were stained with Cadm4, APC and DAPI for imaging (**h**), the fluorescence intensity of Cadm4 and APC was quantified (**i**, two-tailed *t*-test, *n* = 6, **p ≤ 0.01, ****p < 0.0001). **j** PM fractions were prepared from corpus callosum of WT and ZDHHC3-KO mice and analyzed for the levels of Cadms and Mag by WB and quantified (two-tailed *t*-test, *n* = 3, **p ≤ 0.01). Immunoprecipitations were performed with the brain lysates of WT and ZDHHC3-KO mouse by using Cadm4 antibody, the IP eluent was analyzed for Cadm4 binding proteins (**k**), and quantified (**l**, two-tailed *t*-test, *n* = 3, *p ≤ 0.05, **p ≤ 0.01, ***p < 0.001). Data are represented as mean ± SEM

To further extend the evidence that Cadm4 palmitoylation is required for CNS myelination, we reasoned that downregulating palm-Cadm4 by deleting ZDHHC3 may cause similar myelin defects as in Cadm4-KI mouse. For verification, ZDHHC3-KO mouse was generated (Supplementary Fig. 5a–d) and again, the loss of ZDHHC3 significantly reduces the level of palm-Cadm4 in ZDHHC3-KO as compared to that of WT mouse (Fig. 6a, b). For evaluating CNS myelination, images of electron microscopy presented that as the myelinated axons per field of view is significantly downregulated, the percentage of abnormal myelination, e.g., demyelination, thickened myelin sheath/hypermyelination etc. is dramatically upregulated in ZDHHC3-KO as compared to that of the WT mouse (Fig. 6c, d), virtually phenocopies that of the Cadm4-KI mouse (Fig. 4c, d). Consistently, the value of g-ratio is downregulated in ZDHHC3-KO as compared to that of the WT mouse (Fig. 6d), due to the fact of hypermyelination (arrow pointed, Fig. 6c).

Similarly, at cellular level, we could show that MBP expression or its fluorescence intensity is dramatically downregulated in corpus callosum of ZDHHC3-KO as compared to that of WT mouse (Fig. 6e, f), which may account for the defects of myelination (Fig. 6g). Simultaneously, the fluorescence intensity of APC and Cadm4 is significantly reduced in ZDHHC3-KO mouse (Fig. 6h, i), verifying that oligodendrocyte maturation is indeed impaired. At molecular level, the biochemical data showed that the PM localization of Cadm4, as well as other cell adhesion molecules e.g., Cadm2-3 and Mag is significantly decreased in corpus callosum of ZDHHC3-KO as compared to that of WT control (Fig. 6j), which may result in the dissociation of protein-protein binding between Cadm4 and other cell adhesion molecules, but enhance the binding of Cadm4 and Clathrin (Fig. 6k, l). Notably, the treatment of CHQ or Dynasore was able to rescue the expression or PM localization of Cadm4 in either ZDHHC3-KO or Cadm4-KI mice (Supplementary Fig. 5e–h). In summary, the above results strengthened the axis that ZDHHC3 mediated Cadm4 palmitoylation is required for proper oligodendrocyte maturation and myelination in CNS via regulating PM presence of Cadm4 and its association with other cell adhesion molecules.

### Cadm4 palmitoylation is tightly involved in neuroinflammation in models of demyelination

**Fig. 7:**
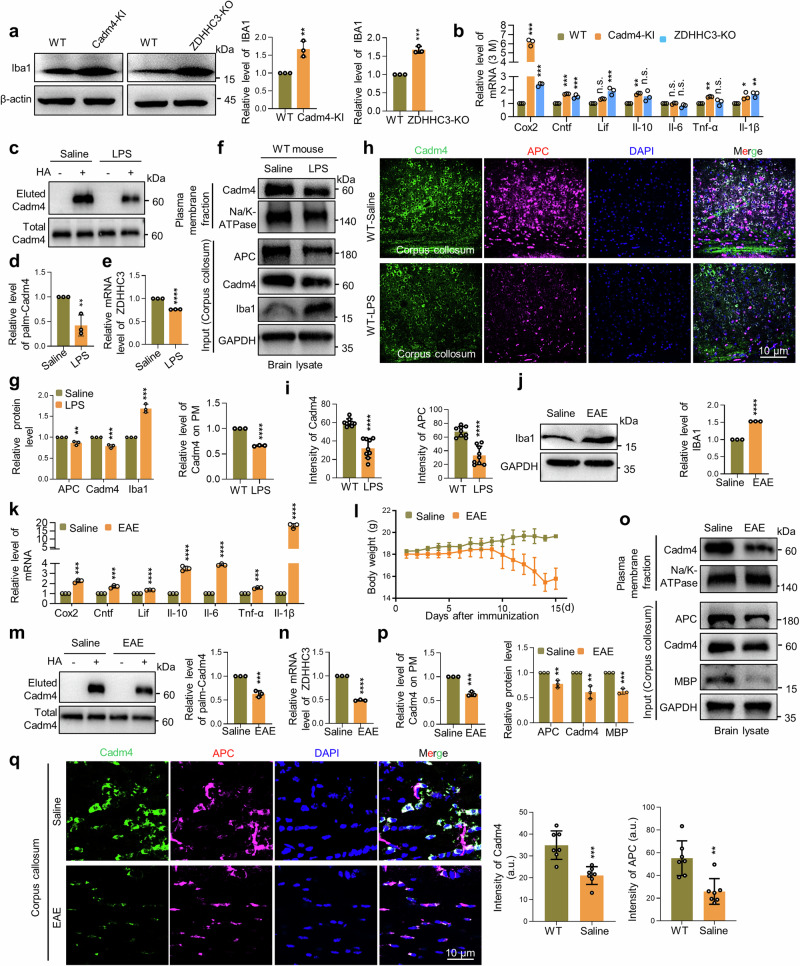
ZDHHC3-Cadm4 signaling is tightly involved in neuroinflammation in models of demyelination diseases. **a** Lysates of corpus callosum from different genotypes were subjected for WB analysis of the level of Iba1 (two-tailed *t*-test, *n* = 3, **p ≤ 0.01, ***p < 0.001). **b** Relative mRNA level of neuroinflammation markers was evaluated by Real-time PCR in corpus callosum of different genotypes (one-way ANOVA followed by Bonferroni post hoc test, *n* = 3, *p ≤ 0.05, **p ≤ 0.01, ***p < 0.001). Saline/LPS-treated mice were sacrificed, the tissue of corpus callosum was evaluated for the levels of palm-Cadm4 (**c**, **d**, two-tailed *t*-test, *n* = 3, **p ≤ 0.01) and ZDHHC3 (**e**, two-tailed *t*-test, *n* = 3, ****p < 0.0001). Total proteins and PM fractions were prepared from corpus callosum of Saline/LPS-treated mice and analyzed by WB (**f**), levels of total protein as well as PM level of Cadm4 were quantified (**g**, two-tailed *t*-test, *n* = 3). Frozen coronal sections of corpus callosum were prepared from LPS/Saline-treated mice and stained with Cadm4, APC and DAPI for imaging (**h**), the fluorescence intensity of Cadm4 and APC was quantified (**i**, two-tailed *t*-test, *n* = 3, ****p < 0.0001). **j** Lysates of corpus callosum from EAE/Saline-treated mice were subjected for WB analysis of the level of Iba1 (two-tailed *t*-test, *n* = 3, ****p < 0.0001). **k** Relative mRNA level of neuroinflammation markers was evaluated by Real-time PCR in corpus callosum of EAE/Saline-treated mice (two-tailed *t*-test, *n* = 3). **l** Progression of body weight was measured in EAE/Saline-treated mice models (paired *t*-test, ***p < 0.001). EAE/Saline-treated mice were sacrificed, the tissue of corpus callosum was evaluated for the levels of palm-Cadm4 (**m**, two-tailed *t*-test, *n* = 3, ***p < 0.001) and ZDHHC3 (**n**, two-tailed *t*-test, *n* = 3, ****p < 0.0001). Total proteins and PM fractions were prepared from corpus callosum of EAE/Saline-treated mice and analyzed by WB (**o**), levels of total protein as well as PM level of Cadm4 were quantified (**p**, two-tailed *t*-test, *n* = 3). **q** Frozen coronal sections of corpus callosum were prepared from LPS/Saline-treated mice and stained with Cadm4, APC and DAPI for imaging, and the fluorescence intensity of Cadm4 and APC was quantified (two-tailed *t*-test, *n* = 7). Data are represented as mean ± SEM

As neuroinflammation is prevalent in varied myelination abnormalities,^25,26^ and to explore the physiological context that may regulate the axis of ZDHHC3-Cadm4 palmitoylation, we examined the microenvironment of corpus collosum in different genotypes and the results clearly suggested a neuroinflammation situation in both ZDHHC3-KO and Cadm4-KI mice (Fig. 7a, b (3 month-old) and Supplementary Fig. 6e (1 month-old)). Therefore, LPS-induced neuroinflammation mouse model was established and interestingly, the results showed that the level of palm-Cadm4 is significantly downregulated in LPS-treated mouse model (Fig. 7c, d), as well as that of the expression of ZDHHC3 (Fig. 7e), suggesting of the alteration of ZDHHC3-Cadm4 signaling. Truly, as consequence, the PM localization of Cadm4 (Fig. 7f, g), the fluorescence intensity of APC and Cadm4 (Fig. 7h, i) are significantly impaired in LPS-treated group as compared to that of the saline-treated group.

Moreover, in another neuroinflammation mouse model e.g., EAE (Experimental autoimmune encephalomyelitis), similar scenario was uncovered: as neuroinflammation is activated (Fig. 7j–l and Supplementary Fig. 6f), the level of palm-Cadm4 as well as the expression of ZDHHC3 are significantly downregulated (Fig. 7m, n), consequently, the PM localization of Cadm4 (Fig. 7o, p), the fluorescence intensity of APC and Cadm4 (Fig. 7q) are significantly impaired in EAE as compared to WT control mouse. These data implied that ZDHHC3-Cadm4 signaling is tightly involved in neuroinflammation, which may well be shared pathological mechanism for varied demyelination diseases related to neuroinflammation.

To further support this notion, a reversible demyelination and remyelination model was utilized. Lysolecithin (LPC)^27^ was injected into the corpus callosum of mice with different genotypes to induce local demyelination (Supplementary Fig. 7a). Notably, demyelination was observed in the corpus callosum by day 7 but showed remyelination by day 21 in WT mice. However, this remyelination process was inhibited in both Cadm4-KI and ZDHHC3-KO mice (Supplementary Fig. 7b). These findings suggest a strong link between Cadm4 palmitoylation and the reversible process of demyelination and remyelination. Specifically, the level of palm-Cadm4 was significantly decreased on day 7 following LPC induction, but returned to normal levels by day 21 in WT mice (Supplementary Fig. 10b). However, this pattern was disrupted in Cadm4-KI or ZDHHC3-KO mice due to the alteration of Cadm4 palmitoylation. Also, it should be noted that microglia and astrocytes either accumulate (day 7) or recede (day 21) at the LPC induction site in WT, but retain on site at day 21 in Cadm4-KI or ZDHHC3-KO mice (Supplementary Figs. 8 and 9), therefore a similar trend of neuroinflammation follows (Supplementary Fig. 10a). Notably, other stimulus as oxidative stress induced by H_2_O_2_ can also manipulate the level of palm-Cadm4 (Supplementary Fig. 10c). Collectively, these findings indicate that neuroinflammation induced in LPS and EAE models can modulate the level of palm-Cadm4, resulting in demyelination. Conversely, the induction of demyelination by LPC can also regulate Cadm4 palmitoylation and neuroinflammation in a reversible manner.

### Cadm4 is predominantly expressed in oligodendrocyte precursor cells and oligodendrocytes and involved in cell differentiation trajectory

To better understand the role of Cadm4 and its palmitoylation in different cell types within the brain, we collected samples of the hippocampus and corpus callosum from all genotypes for single-nucleus RNA sequencing (snRNA-seq, Supplementary Fig. 11a). The data indicate that Cadm4 is primarily expressed in oligodendrocyte precursor cells (OPCs) and oligodendrocytes, with weaker expression in astrocytes and neurons, but not in microglia (Supplementary Fig. 11b). Upon quantifying various cell populations, it became evident that the population of microglia is expanded in Cadm4-KI and ZDHHC3-KO mice compared to WT (Supplementary Fig. 11c). Further analysis confirms the increased population or activation of microglia and astrocytes in Cadm4-KI and ZDHHC3-KO mice compared to WT (Supplementary Fig. 11d).

Given that Cadm4 is mainly expressed in OPCs and oligodendrocytes, we conducted CytoTRACE analysis to evaluate cell differentiation status and GCS analysis to assess gene set expression profiles. The CytoTRACE analysis indicates a less differentiated state in both OPCs and oligodendrocytes in Cadm4-KI and ZDHHC3-KO mice compared to WT (Supplementary Fig. 11e). Additionally, the GCS analysis shows variations in gene set expression profiles (Supplementary Fig. 11f), highlighting the impact of manipulating Cadm4 palmitoylation on cell differentiation trajectories. These findings suggest that changes in Cadm4 palmitoylation levels may disrupt the normal differentiation process of OPCs and oligodendrocytes, potentially affecting myelination in the CNS.

Furthermore, an analysis of the snRNA-seq data within the neuronal pool reveals differences in a specific cluster of neurons among the various genotypes (Supplementary Fig. 12a). KEGG analysis uncovers alterations in axon guidance, cell adhesion molecules, long-term potentiation, and synaptic functions in Cadm4-KI or ZDHHC3-KO genotypes compared to WT (Supplementary Fig. 12a, b). However, these functional changes in neurons cannot solely be attributed to Cadm4 and its palmitoylation given their minimal expression levels. Therefore, these data underscore the critical role of Cadm4 in regulating the differentiation trajectory of OPCs and oligodendrocytes, thereby influencing CNS myelination.

Briefly, to explore the potential mechanism underlying Cadm4-regulated oligodendrocyte differentiation, we screened related canonical signaling pathways (NOTCH/WNT-β-Catenin/IGF/GPR17-ID2) in the corpus callosum of mice at both postnatal day 1 (P1) and 2 months of age (2 M). Interestingly, the results showed that the WNT-3β-Catenin pathway was prominently disrupted at both time points in both Cadm4-KI and ZDHHC3-KO genotypes relative to WT (Supplementary Fig. 13a). To validate these findings at the protein level, we confirmed substantial upregulation of WNT3a and β-Catenin in both mutant genotypes compared to WT (Supplementary Fig. 13b, c). Furthermore, to understand the upregulation of β-Catenin, we examined the upstream regulators controlling its protein stability. The data demonstrated a notable increase in both p-GSK3β and p-AKT levels, and a decrease in PTEN level in both Cadm4-KI and ZDHHC3-KO genotypes compared to WT (Supplementary Fig. 13b, c), implying modulation of the AKT-GSK3β signaling axis. As stabilized β-Catenin may translocate to the nucleus and suppress oligodendrocyte maturation, we examined its subcellular distribution. Indeed, β-Catenin levels were notably elevated in both cytoplasmic and nuclear fractions in both mutant genotypes compared to WT (Supplementary Fig. 13d). Consequently, key lineage markers (Oligo1/Myrf/Mbp/Plp) for OPC/oligodendrocytes were significantly downregulated in both Cadm4-KI and ZDHHC3-KO genotypes compared to WT (Supplementary Fig. 13e).

### Altered Cadm4 palmitoylation impairs neuronal transmission and cognitive behaviors in mice

To assess the cellular consequences in neurons resulting from altered levels of Cadm4 palmitoylation, we employed electrophysiology. The results revealed a reduction in the amplitude (Supplementary Fig. 14a–c) and frequency (Supplementary Fig. 14d, e) of spontaneous EPSCs in Cadm4-KI and ZDHHC3-KO mice compared to WT, indicating that diminished levels of palm-Cadm4 significantly impair excitatory neuronal transmission due to demyelination. Furthermore, when we evoked EPSCs by stimulating the presynaptic input fiber, similar results were obtained, suggesting weakened nervous conduction (Supplementary Fig. 14f).

Additionally, to evaluate the physiological consequences in mice resulting from altered levels of Cadm4 palmitoylation, various behavioral tests^28^ were conducted. The results demonstrated that both Cadm4-KI and ZDHHC3-KO mice exhibited impairments in learning and memory (Supplementary Fig. 15e, g, l, n), as well as altered behavior in anxiety-like tasks (Supplementary Fig. 15c, d, j, k) when compared to WT mice. Taken together, these findings imply that altered levels of Cadm4 palmitoylation impair neuronal transmission and lead to defects in cognitive behaviors.

## Discussion

**Fig. 8:**
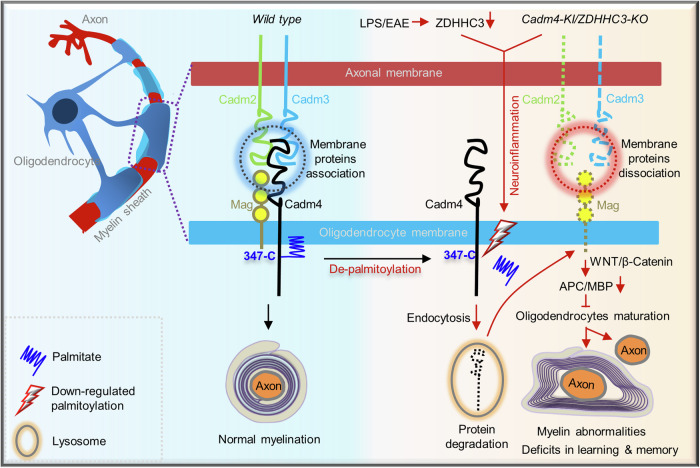
The proposed mechanism that ZDHHC3 mediated palmitoylation regulates CNS myelination via controlling stable PM localization and protein stability of Cadm4. Cadm4 is palmitoylated at cysteine-347 to sustain its stable presence on PM, where it binds with Cadm2/3 and Mag and together, they ensure the proper formation of CNS myelination. Upon the downregulation of palm-Cadm4, either by Cadm4-C347A mutation or the deletion of ZDHHC3, Cadm4 is prone to be internalized and targets for protein degradation, which impairs the interactions of surface proteins and downregulates the level of APC/MBP, the latter may hinder the maturation of oligodendrocytes, potentially through the WNT/β-Catenin signaling pathway, and thus contribute to myelin abnormalities, e.g., loss of myelination, demyelination, and hypermyelination and cognitive deficits in CNS. Most interestingly, ZDHHC3-Cadm4 signaling is tightly involved in neuroinflammation in mice models of demyelination, as downregulating palm-Cadm4 in Cadm4-KI/ZDHHC3-KO mice leads to immuno-activation and neuroinflammation and remarkably, LPS/EAE-induced neuroinflammation significantly downregulates levels of palm-Cadm4 and ZDHHC3, which subsequently modulates PM localization of Cadm4 and deteriorates oligodendrocytes maturation. Combined, the axis of ZDHHC3-Cadm4 palmitoylation is not only vital for CNS myelination but also may serve as conserved pathological mechanism for demyelination diseases related to neuroinflammation

Although the downregulation of Cadm4 is a marked feature in demyelination scenarios,^7,8,13^ the underlying molecular mechanism is unclear. Here, we showed that ZDHHC3 mediated palmitoylation is required for the stable PM localization of Cadm4, reducing Cadm4 palmitoylation by either downregulating or the depletion of ZDHHC3 renders the internalization of Cadm4 from PM and targets for protein degradation (Figs. 1–3 and 7). Regarding the axon-glia contact mediated by Cadms and Mag on PM are vital for myelination,^7–10,13,29,30^ downregulated Cadm4 impairs its association with Cadm2/3 and Mag. This impairment substantially hinders the maturation of oligodendrocytes, potentially through the WNT/β-Catenin signaling pathway, resulting in myelin abnormalities e.g., loss of myelination, demyelination or hypermyelination and functional deficits in CNS (Figs. 4–6). Critically, it was shown that ZDHHC3-Cadm4 signaling is tightly involved in neuroinflammation in different models of demyelination diseases (LPS/EAE/LPC, Fig. 7 and Supplementary Figs. 7–10), emphasizing not only the key roles of ZDHHC3-Cadm4 axis for myelination but also a conserved pathological mechanism for varied demyelination diseases (Fig. 8a).

During myelination, Oligodendrocytes send multiple exploratory cellular processes that contact numerous axons, some stabilize and develop into myelin sheath while the others retract,^5,31^ indicating that myelination is a dynamic process and it is plausible that the underlying machinery regulating myelination may adapt to this dynamicity as well. Indeed, we presented in principal that ZDHHC3-regulated Cadm4 palmitoylation (Fig. 5), a reversible process, is engaged in controlling the formation of myelin sheath in CNS in both Cadm4-KI and ZDHHC3-KO mice (Figs. 4 and 6). Additionally, the snRNA-seq data show that Cadm4 is predominantly expressed in OPCs and oligodendrocytes, and the CytoTRACE and GCS analysis highlights the impact of Cadm4 palmitoylation manipulation on cell differentiation trajectories, suggesting that alterations in Cadm4 palmitoylation levels may disrupt the normal differentiation process of OPCs and oligodendrocytes, thus likely influencing myelination. Indeed, further investigation into the potential mechanism underlying Cadm4-regulated oligodendrocyte differentiation suggests that upregulation of WNT may activate the downstream AKT-GSK3β signaling axis. Increased p-GSK3β deactivates itself,^32^ leading to cytoplasmic β-Catenin stabilization and subsequent nuclear translocation (Supplementary Fig. 13), potentially inhibiting OPC/oligodendrocyte differentiation^33,34^ in both Cadm4-KI and ZDHHC3-KO mice. While we should not overlook the possibility that other pathways may also be involved, considering the alterations in NOTCH/IGF/GPR17-ID2 levels,^32^ key lineage markers (Oligo1/Myrf/MbP/Plp)^32^ for OPC/oligodendrocytes were significantly downregulated in both Cadm4-KI and ZDHHC3-KO genotypes. This reinforces the notion that attenuated Cadm4 palmitoylation suppresses OPC/oligodendrocyte differentiation.

Considering that the deletion of ZDHHC3 partially downregulates the level of palm-Cadm4 (Figs. 5a and 6a), implying that other ZDHHCs might be also involved in palmitoylating Cadm4. Nevertheless, the phenotype of similar myelin abnormalities developed in both Cadm4-KI and ZDHHC3-KO mouse (Figs. 4 and 6) suggests that ZDHHC3 is critical for the maintenance of appropriate level of palm-Cadm4 in mouse brain. Consistently, in other demyelination mouse models, the axis of ZDHHC3-Cadm4 palmitoylation is repeatedly seen to be modulated (Fig. 7c–e, m, n), which may result in defects in the maturation of oligodendrocytes and thus the demyelination pathology. Additionally, to complete the cycle of palmitoylation and depalmitoylation, it might be interesting to determine which enzyme (APT1/2, PPT1/2 and ABHD17a) might catalyzes Cadm4 depalmitoylation in CNS.

By analyzing palmitoylated proteins containing single/multiple TM domain(s), it is found out that these proteins tend to be modified at the cysteine residue close to the TM or cytoplasmic boundary,^35–39^ interestingly, such rule is applicable to Cadm4 as Cadm4 is palmitoylated at cysteine-347, a region right after the TM domain and in the beginning of the cytoplasmic tail (Fig. 1d). Palmitoylation at such specific site might have different functional implications, e.g., association with phospholipids or changing the conformation of TM domain,^40–42^ which might interfere protein-protein interaction, signaling transduction or protein stability.^42,43^ In agreement with the latter, our results showed that reducing the level of palm-Cadm4, either by mutation (Cadm4-C347A) or the removal of ZDHHC3 (ZDHHC3-KO), promotes PM internalization of Cadm4 and targets for protein degradation via autophagy-lysosome pathway (Figs. 3 and 5d–h), which in return regulates the level of Cadm4 on PM. Surprisingly, palmitoylation on other proteins might have opposite effect. It was shown that AMPA receptors are also palmitoylated at the TM/cytosol boundary, upon palmitoylation, AMPA receptors decrease its interaction with cytoskeleton and therefore tend to be internalized and dissociate from PM.^36^ Additional evidences demonstrated that palmitoylation at such site is involved in diversified regulations e.g., subcellular trafficking or protein-protein interaction, etc.,^35,36,38,40^ suggesting that the roles of palmitoylation at TM/cytosol boundary might vary depending on the proteins investigated but the cysteine residue locates at the TM/cytosolic boundary might be a hotspot for protein palmitoylation.

Interfering Cadm4 palmitoylation, either in Cadm4-KI or ZDHHC3-KO mouse, induces similar myelination abnormalities in CNS, resembling part of the Charcot-Marie-Tooth (CMT) neuropathy,^2,7,13,30^ suggesting that Cadm4 might be a key substrate of ZDHHC3 in regulating CNS myelination although this cannot rule out other possibilities. It might be logic to understand the loss-of-myelination phenotype, as less-palmitoylated Cadm4 reduces its presence on PM (Figs. 4j and 6j), weakens its interactions with Cadm2/3 and Mag (Figs. 4k and 6k) and hence inhibits myelination or leads to detachment of the axon from the myelin sheath, a scenario was demonstrated constantly in circumstances where either their expression levels or protein-protein interactions were manipulated.^6,7,9,13,29,30^ Apart from that, other myelin abnormalities as thickened myelin sheath in ZDHHC3-KO/Cadm4-KI mouse might be interpreted by altered molecular signaling in guiding myelination. It has been suggested that myelination downstream of erbB2 is controlled by local phosphoinositide production,^44,45^ the axonal signaling molecule neuregulin1 type III^46,47^ stabilizes Dlg1 and PTEN complexes by inhibiting their protein degradation, PTEN dephosphorylates PtdIns3,4,5 and antagonizes AKT/PI3K activation (by reducing AKT phosphorylation) downstream of erbB2.^48^ Notably, the loss of PTEN in myelinating Schwann cells leads to myelin overgrowth as focal hypermyelination and tomaculous neuropathy.^45^ Furthermore, it was reported that Cadm4 recruits Par3, a multiple-PDZ domain containing protein, to the Schwann cell adaxonal membrane,^49^ and Par3 interacts with PTEN and regulates cell polarity.^50^ Interestingly, we did show that AKT phosphorylation is upregulated in the brains of ZDHHC3-KO and Cadm4-KI as compared to that of the control mouse (Supplementary Fig. 6a–d), hinting that AKT signaling might be involved in promoting the overgrowth of myelin sheath. However, the most part of the regulatory machinery relating palm-Cadm4, AKT signaling and diversified myelin abnormalities remains elusive and invites future studies.

To understand the physiological context in which ZDHHC3-Cadm4 palmitoylation might be modulated, we analyzed various models of neuroinflammation and demyelination. Interestingly, our data showed that the levels of palm-Cadm4 and ZDHHC3 are downregulated in both LPS and EAE models, suggesting that ZDHHC3-Cadm4 signaling may represent a conserved pathological mechanism underlying various demyelination diseases. Additionally, it is worth noting that other stimuli, such as oxidative stress induced by H_2_O_2_, can also influence the level of palm-Cadm4, indicating that a broader range of physiological stimuli may be involved.

The results from LPC demyelination, LPS, and EAE models indicate a bidirectional relationship between Cadm4 palmitoylation, myelination, and neuroinflammation in the CNS. In the LPC model, damage to myelination affects palm-Cadm4 levels first, triggering immune responses. Conversely, in LPS and EAE models, immune response activation leads to downregulated palm-Cadm4, showing a mutual correlation. Studies on Cadm4-KI and ZDHHC3-KO mice during LPC induction reinforce this bidirectional link, with disrupted Cadm4 palmitoylation sustaining neuroinflammation. snRNA-seq analysis reveals the role of Cadm4 in OPCs and oligodendrocytes, impacting myelination trajectories. Increased microglia and astrocyte populations in Cadm4-KI and ZDHHC3-KO mice support the impact of Cadm4 on myelination and CNS astrocyte/microglia status. This complex interplay underscores the bidirectional relationship among Cadm4 palmitoylation, myelination, and neuroinflammation in CNS.

Collectively, our findings reveal a regulatory mechanism that the stable presence of Cadm4 on PM is regulated by protein palmitoylation mediated by ZDHHC3, which is required for proper myelination and cognitive behaviors in CNS. Moreover, the axis of ZDHHC3-Cadm4 palmitoylation may underlie varied types of demyelination diseases related to neuroinflammation and thus a potential target for intervention.

## Materials and methods

### Mouse models and ethics statements

All animals are kept in SPF environment which is a temperature-controlled room with a 12 h light/dark cycle, water and food are adequately supplied. All animal procedures were performed according to guidelines approved (Certificate No: XYLL-20210179) by the committee on animal care at Xinxiang Medical University. For all experiments, both male and female littermates were randomly assigned and the principal of double-blind was adopted in all experiments and the following data analysis.

### Generation of Cadm4-KI mouse

C57BL/6 (B6) mouse were purchased from Beijing Vital River Laboratory Animal Technology. Fertilized B6 eggs were collected and injected via microinjection system as previously described with little modification. In brief, Cas9 mRNA and sgRNA (TCACTTACCCTTCTGTCGGACGG, TGGTGCTCCGTCCGACAGAAGGG) were produced by using in vitro transcript (IVT) kits, the single-stranded oligonucleotide donor DNA (ssODN) was synthesized by Bioligo company (Shanghai, China), all these components were mixed and microinjected into the cytoplasm of fertilized eggs. Injected eggs were cultured to two-cell stage and then transferred into ICR foster mouse. Twenty-days later, F0 mouse were born and genomic DNA was isolated. The primers: F: TAGAGGCGTGTAGATACCAGGAGTGAA and R: GCTAAGACAGAGTCTATCAATGGAGTCCC were used for genotyping.

### Generation of ZDHHC3-KO mouse

Similarly, Cas9 mRNA and sgRNA (AACTCTGCCTCCACGAGGCA GGG, CCGACACAGTTGTTGACCCA AGG) were produced by using in vitro transcript (IVT) kits, all these components were mixed and microinjected into the cytoplasm of fertilized eggs. Injected eggs were cultured to two-cell stage and then transferred into ICR foster mouse. Twenty-days later, F0 mouse were born and genomic DNA was isolated. The primers: F: TGATGGTTACCCCTGCTGAA and R: TTCTCAACAGCAACCAAGACT were used for genotyping. Total mRNA was isolated for qRT-PCR, and the primers: F: CCACCCATCACTTCCGAGAC and R: CAGGTGACAATGGCACAAGC were used for the transcriptional quantification of ZDHHC3.

### Generation of ZDHHC3-KO cells in N2a

As N2a is a mouse cell line, we applied the same knockout strategy for N2a as carried out in ZDHHC3-KO mouse. Briefly, two guide RNAs (AACTCTGCCTCCACGAGGCAGGG, CCGACACAGTTGTTGACCCAAGG) were synthesized and cloned into pX458 vector with EGFP which enabled single cell sorting. N2a cells were co-transfected with plasmids of px458-sgRNA1 and px458-sgRNA2 by Lipofectamine 3000 reagent with the reduced serum medium Opti-MEM. Two days later, single cell was sorted by BD FACSAria™ Fusion and then cultured until the formation of colonies. Individual colonies were screened by PCR and sanger sequencing was performed for further verification. N2a was maintained in MEM medium (Gibco, USA) supplemented with 10% fetal bovine serum (Gibco, USA), 100 μg/ml streptomycin, 100 U/ml penicillin, 1 mM sodium pyruvate and 1% glutamine (100×, Gibco), and incubated in 5% CO_2_ at 37 °C.

### Plasmids

The plasmids expressing mouse-origin Cadm4-Flag, Cadm4-GFP, APT1-Flag, APT2-Flag, PPT1-Flag, PPT2-Flag and ABHD17a-Flag were constructed. The cDNA for the mouse Cadm4, APT1, APT2, PPT1, PPT2 or ABHD17a was obtained by RT-PCR which was then subcloned into pUC19 as cDNA donors. Specific primers were synthesized for subcloning individual cDNA into pCMV3-C-Flag for mammalian cell expression by In-Fusion cloning method. The Cadm4-C339A-Flag, Cadm4-C347A-GFP, Cadm4-C347A-Flag, Cadm4-C339/347A-GFP and Cadm4-C339/347A-flag mutants were generated by site-directed mutagenesis PCR reaction. Other plasmids as RFP-Rab5 (Plasmid 14437), RFP-Rab7 (Plasmid 14436) and RFP-Lamp1 (Plasmid 1817) were obtained from Addgene. All plasmids of Ha-DHHCs were a gift from Dr. Fukata. All constructs were verified by sequence analysis.

### Cell culture, transfection and treatments

HEK-293T (CRL-11268) and N2a (CCL-131) were obtained from ATCC. HEK-293T and N2a were grown in DMEM high glucose medium (Gibco, USA) and MEM (Hyclone) respectively, supplemented with 10% fetal bovine serum (Gibco, USA) containing 100 μg/ml streptomycin and 100 U/ml penicillin at 37 °C in a 5% CO_2_ incubator. All cell lines were transfected using Lipofectamine 3000 reagent for 24 h (Invitrogen) with the reduced serum medium Opti-MEM (Life Technologies). The follow reagents were used for cells treatments: Dimethyl sulfoxide (DMSO) (Sigma-Aldrich, Cat# D8418), 50 μM 2-Bromohexadecanoic acid (2-BP) (Sigma-Aldrich, Cat# 238422), 100 μM Cycloheximide (CHX, MedChemExpress, HY-12320), 25 μM Mg132 (MedChemExpress, HY-13259), 50 μM Chloroquine (CHQ, Sigma, C6628), 3 mM 3-Methyladenine(3-MA, MedChemExpress, HY-19312) and 80 μM dynasore (MedChemExpress,HY-15304). All drug experiments were started at 12 h after cells were transfected. Cells were exposed to fresh MEM complete medium containing drugs or control DMSO for the indicated time and then washed with PBS for 3 times.

### Acetyl-resin-assisted capture assay (Acyl-RAC)

RAC assay was performed as previously described with minor modification (NEM was used to replace MMTS). Briefly, mouse brain/cells were lysed in lysis buffer (20 mM Tris pH = 7.5, 150 mM NaCl, 1% Triton X-100) containing protease inhibitor cocktail (Roche). Lysate was sonicated and incubated at 4 °C for 30 min while rotating, which was then cleared by centrifugation at 15,000 × *g* for 10 min at 4 °C, the supernatant was harvested. The amount of protein was quantified with a bicinchoninic acid (BCA) assay Kit (Cat#P0009, Beyotime). Protein lysate (1 mg) was diluted to 1 mg/ml with blocking buffer (100 mM HEPES, 1 mM EDTA, 2.5% SDS, 50 mM NEM, pH 7.5) and kept at 50 °C for 60 min with agitation. NEM was removed by four sequential 70% acetone precipitations, pellet was resuspended in 600 μl binding buffer (100 mM HEPES, 1 mM EDTA, 1% SDS, pH 7.5). Samples were divided into two equal parts and added with 50 μl pre-equilibrated thiopropyl Sepharose 6B (Cat#17-0420-01, GE Healthcare) respectively, one part was treated with 40 μl 2 M hydroxylamine (HA+) pH 7.0 (to cleave thioester linkage) and the other part was incubated with 40 μl 2 M NaCl (negative control, HA−), 20 μl of each supernatant was taken as “input”. Cleavage and capture were carried out on a rotator at room temperature for 4 h. Resins were washed five times with binding buffer, eluted with 40 μl Laemmli loading buffer (2.1% SDS, 66 mM Tris-HCl (PH7.5), 26% (W/V) glycerol, 50 mM DTT) on a shaker at 42 °C for 15 min and subjected for Western blot analysis.

### mPEG-labeling assay

mPEG-labeling was performed as described previously. Cultured cells/tissues were lysed with 4% SDS in TEA buffer (50 mM Triethanolamine, 150 mM NaCl, pH = 7.3) supplemented with protease inhibitor cocktail (Roche). Total 500 μg protein was treated with 10 mM TCEP for 30 min and then incubated with 25 mM NEM for 2 h at room temperature to block unmodified cysteine residues. NEM blocking was terminated by 3-times methanol-chloroform-H_2_O precipitation (4/1.5/3) and dried in a speed-vacuum (Centrivap Concentrator, Labconco) after the last precipitation to remove NEM completely. The pellet was resuspended in 100 μl TEA buffer containing 4% SDS and incubated at 37 degree for 10 min with gentle agitation. Next, the samples were treated with 1 M neutralized NH2OH at room temperature for 1 h to cleave the thioester linkage formed between cysteine residue and palmitate, which were then subjected for methanol-chloroform-H_2_O precipitation and resuspended in 30 μl TEA buffer containing 4% SDS and 4 mM EDTA at 37 degree for dissolving. For mPEG-maleimide alkylation, additional solution containing 0.2% Triton X-100, 90 μl TEA buffer and 1.33 mM mPEG-Mal (final concentration at 10 mM) was added and incubated at room temperature for 2 h. Last, the reaction was stopped by a final methanol-chloroform-H_2_O precipitation and resuspended in laemmli buffer (Bio-Rad) for WB analysis.

### Immunofluorescence staining and imaging

N2a cells were plated onto poly-D-lysine-coated coverslips and transfected as described with indicated plasmids. At 24 h post-transfection, the cells were fixed with 4% (W/V) paraformaldehyde (Electron Microscopy Sciences, Cat#15710). After blocking in 3% BSA in PBS, cells were stained (without permeabilization) with primary antibody Cadm4 (abcam, Cat# ab69605, 1:500), Na/K ATPase (Abcam, Cat# ab76020, 1:300), Flag-M2 (sigma, F3165, 1:1000) and then incubated with secondary antibodies Anti-Rabbit IgG (H + L) Alexa Fluor Plus 488 conjugated (Invitrogen, Cat#A11070, 1:1000), Anti-Rabbit IgG (H + L) Alexa Fluor Plus 594 conjugated (Invitrogen, Cat#A11072, 1:1000) and Alexa Fluor Plus 647 conjugated (Invitrogen, Cat#A21245, 1:1000), washed, and mounted onto slides with Fluoromount-G (Electron Microscopy Sciences, Cat#17984-25). The fluorescence images were taken with a Stimulated Emission Depletion microscopy (Leica TCS SP8 STED).

### Live-cell imaging

N2a cells were plated onto glass-bottom dish (Eppendorf, Cat#0030740017) and transfected with Cadm4-GFP/RFP-Rab5 or C347A-GFP/RFP-Rab5, after 15 h, live-cell imaging was taking for about 30 min (Leica TCS SP8 STED). Fluorescence intensity as well as Pearson’s coefficient were analyzed by LAS X software associated with SP8 STED.

### Plasma membrane fractionation and nucleus fractionation

N2a cells/tissues were homogenized in HME buffer (10 mM HEPES, 1 mM MgCl2, 1 mM EDTA, PH7.4) supplemented with protease inhibitor cocktail (Roche). The homogenates were then subjected for Freeze/Thaw cycle (freeze in liquid nitrogen, thaw at 37 °C) for 5 times, and then sonicated 10 s on/off for 1 min on ice using a microsonicator (Scientz, UP-250). The homogenates were centrifuged at 900 × *g* for 10 min to remove cell debris and nuclei, the supernatant was collected and centrifuged at 20,000 × *g* for 40 min at 4 degree and the supernatant was collected as cytosol fractions. The pellets (P1) were resuspended in HEM buffer (with protease inhibitor) and centrifuged at 10,000 × *g* for 30 min, 4 degree. The pellets (P2) were collected as subcellular organelle membrane fractions, and the supernatant was subjected for last centrifugation at 20,000 × *g* for 40 min, 4 degree, the pellets (P3) were collected as plasma membrane fractions (majority). For verification, all fractions were examined by Western blot and their corresponding marker proteins (HSP-90 and Na/K ATPase were used as markers for cytosol and plasma membrane fractions respectively). Nuclear fractions were isolated using a Nuclear Protein Extraction Kit (Beyotime, P0028) following the manufacturer’s protocol.

### Western blot analysis and antibodies

Samples were separated in standard SDS-PAGE gels and transferred to Immobilon-P PVDF membrane (Pore size 0.2 μM, EMD Millipore). The membrane was then blocked in 5% (w/v) skimmed milk in TBS containing 0.1% (v/v) Tween-20 (TBST) for 90 min. After blocking the membranes were washed in TBST and incubated with primary antibody for overnight at 4 °C. After washing with TBST, the membranes were incubated with a suitable horseradish peroxidase (HRP) labeled secondary antibody and signals were detected with an ECL kit (Tanon). The following primary antibodies were used: The following primary antibodies were used: Cadm4 (Abcam, Cat# ab69605, 1:1000), Cadm2 (Abcam, Cat# ab64873, 1:1000), Cadm3 (Abcam, Cat# ab69604, 1:1000), Na/K-ATPase (Abcam, Cat# ab76020, 1:20,000), HSP90 (Cell Signaling, Cat# 4874S, 1:1000), LaminA (Abcam, Cat# ab26300, 1:1000), MAG (Cell Signaling Technology, Cat# 9043S, 1:1000), Flag (Abclonal, Cat# AE024, 1:5000), WNT3a (HUAbio, Cat# HA500193, 1:1000), β-Catenin (Servicebio, Cat# GB11015, 1:1000), Phospho-GSK3β (Ser9) (Cell Signaling Technology, Cat# 5558S, 1:1000), GSK3β (Cell Signaling Technology, Cat# 12456S, 1:1000), Phospho-AKT (Cell Signaling Technology, Cat# 9271S, 1:1000), AKT (Cell Signaling Technology, Cat# 2920S, 1:1000), PTEN (Abcam, Cat# ab267787, 1:1000), Actin (Abcam, Cat# ab7817 1:10000) and Gapdh (Abclone, Cat# AC033, 1:5000). The following secondary antibodies were used for immunoblot: Goat Anti-Rabbit IgG (H + L), HRP Conjugate (Protein Biotechnologies, Cat# PMS302, 1:5000) and Goat Anti-Mouse IgG (H + L), HRP Conjugate (Protein Biotechnologies, Cat# PMS301, 1:5000).

### Immunoprecipitation

Briefly, cell/tissue lysates were incubated with corresponding antibody (anti-Cadm4, 1:200) or IgG and kept for rotation at 4 degree overnight. The next day, protein A/G beads, which was pre-equilibrated in PBS (pH 7.4), was added and kept for additional 2 h at 4 degree in rotating. The beads were then collected at 1000 × *g* for 2 min and washed at least 3 times with lysis buffer coupled with protease inhibitor cocktail (Roche). After last wash and centrifugation, the beads were eluted with 1 × SDS-PAGE Loading Buffer (Beyotime, Cat#P0015L), heated at 100 degree for 5 min and subjected for Western blot analysis.

### Sample preparation and imaging for electron microscopy

Anesthetized mouse were perfused with normal saline followed by perfusion with freshly prepared fixative containing 4% paraformaldehyde and 2.5% glutaraldehyde supplied with 0.1 M sucrose in 0.1 M phosphate buffer (PB), pH 7.4. The brains were chopped to 1 mm3 blocks, kept for 2 h at room temperature, and then stored overnight at 4 °C. After washing four times with 0.1 M PB, the samples were post-fixed in 2% OsO4/1.5% potassium ferrocyanide for 1 h at room temperature. Then the samples were rinsed several times in ddH2O and en bloc stained with 2% aqueous uranyl acetate overnight at 4 °C. Following several washes in ddH2O, specimens were dehydrated in a series of ethanol solution (30%, 50%, 70%, 85%, 95%, 100%, 100%; 10 min each) and replaced with pure acetone two times for 10 min each. Tissue samples were gradually permeated and embedded with EMbed 812 resin (Electron Microscopy Sciences). Resin blocks were sectioned with a diamond knife (ultra 35°, Diatome, Switzerland) using an ultramicrotome (UC7, Leica Microsystem) and collected in copper grids with a single slot. Ultra-sections (70 nm) were stained with uranyl acetate and lead citrate and examined under electron microscopes Tecnai G2 Spirit (Thermo Fisher Scientific) or Hitachi HT7700 equipped with a CCD camera (Orius 832, Gatan) at 120 kV. EM micrographs were analyzed by ImageJ (version 1.50) for myelin thickness and axon diameter. G-ratio was calculated by dividing the inner axonal diameter by the outer axonal diameter. Around 300 axons from 3 mouse (at around P90) were randomly chose for quantification for each genotype.

### Transparency and staining of mouse brain sections

Transparency was performed as previously described.^15^ Briefly, Mice were anesthetized and sacrificed by intracardiac perfusion with 4% paraformaldehyde, then mice brains were excised and post-fixed with 4% paraformaldehyde for no more than 24 h. Sections of 100 μm were cut on leica VT1200 and incubation with hydrogel for 24 h at 4 °C, followed by polymerization for 3 h and electrophoresis for 1 h to remove lipids at 37 °C. For staining, following antibodies were used: Cadm4 (R&D Systems, MAB41641, 1:100); APC (Abcam, ab16794; 1:200); Iba1 (Abclonal, A19776; 1:100), GFAP (Abcam, ab7260; 1:100), MBP (Abcam, ab40390; 1:100) and NF (Abcam, ab7794; 1:100), Alexa Fluor-488 (Invitrogen, A11006; 1:200), Alexa Fluor-594 (Invitrogen, A11037; 1:200) and Alexa Fluor-647 (Invitrogen, A21236; 1:200). Fluorescence intensity as well as Pearson’s coefficient were analyzed by LAS X software associated with SP8 STED. The percentage of myelination was analyzed manually based on the colocalization of MBP/NF.

### Lipopolysaccharides (LPS) treatment

Adult mice at age of 10–12 weeks were injected intraperitoneally with lipopolysaccharides (SIGMA Cat #L2880) at a dose of 5 mg/kg, once a week, three injections at a time, and one injection every hour. The same dose of normal saline was injected as a control group. Four weeks later, mice were sacrificed for other experiments.

### Experimental autoimmune encephalomyelitis (EAE)

Female mice (8–10 weeks) were used. EAE was induced by subcutaneous immunization with 100 μg MOG_35-55_ (synthetized myelin oligodendrocyte glycoprotein residues 35–55, purchased from ChinaPeptides Co., Ltd) peptide emulsified in Complete Freund´s Adjuvant containing 400 μg mycobacteria and intravenous injection of 240 μg pertussis toxin (purchased from MedChemExpress Co., Ltd) in 200 μL dPBS on day 0 and day 2 after immunization.^51^ Experimental mouse was sacrificed at day 15 for analysis.

### Frozen sections and immunohistochemistry

Perfused mouse brain was dissected and post-fixed in 4% PFA for 24 h at 4 °C, then dehydration in 20% and 30% sucrose in turn overnight at 4 °C, optimal cutting temperature compound (OCT) embedding and rapid freezing, and then serial frozen coronal sections (40 μm) were cut on a cryostat (Leica CM1950) and all sections were laid onto coated glass slides and stored at −80 °C. For immunofluorescent staining, frozen slides were incubated in a 37 °C oven for 10–20 min to control the moisture, and then blocking with 3% BSA containing 0.1% Triton X-100 in PBS at 37 °C for 1 h, follow antibodies were used: Cadm4 (R&D Systems, MAB41641, 1:100), APC (Abcam, ab16794; 1:200), Alexa Fluor-488 and Alexa Fluor-647.

### Real-time PCR

Total mRNA was isolated using RNeasy Plus Mini Kit (QIAGEN, Cat# 74134). Then, first-strand cDNA was synthesized by Prime SCRIPT RT Master Mix (TAKARA, Cat# RR036A) and Real-time PCR were conducted on Roche Cobas z480 using TB Green qPCR Premix (TAKARA, Cat# RR820A). Primers were derived from previous research as following: Il-1β: (GATGATAACCTGCTGGTGTGTGA, TTTGTCGTTGCTTGGTTCTCC), Il-6: (GACAAAGCCAGAGTCCTTCAGAGAGATACAG, TTGGATGGTCTTGGTCCTTAGCCAC), Tnf-α: (CCACCACGCTCTTCTGTCTACTG, GCCATAGAACTGATGAGAGG), Il10: (GCTGGACAACATACTGCTAACCG, CCTTGCTCTTATTTTCACAGGGG), Cox2: (CCACTTCAAGGGAGTCTGGA, AGTCATCTGCTACGGGAGGA), Cntf: (GACCTGACTGCTCTTATGGAATCT, GCCTGGAGGTTCTCTTGGA), Lif: (AAACGGCCTGCATCTAAGG, AGCAGCAGTAAGGGCACAAT), Wnt3a: (CACCACCGTCAG[1]CAACAGCC, AGGAGCGTGTCACTGCGAAAG), β-Catenin: (ATCACTGAGCCTGCCATCTG, GTTGCCACGCCTTCATTCC), Notch2: (GAGAAAAACCGCTGTCAGAATGG, GGTGGAGTATTGGCAGTCCTC), Id2: (GCAGCACGTCATCGTCATCGATTACA, TTCAGATGCCTGCAAGGACA), Gpr17: (CTGCTACCTGCTGATCATTCG, TAGACTGAACGGTGGATGTGG), Igf1r: (ATGGCTTCGTTATCCACGAC, AATGGCGGATCTTCACGTAG), Oligo1: (TCATCCTCATCCTCATCCTCTTCC, GCTGCTGCTGTTCCTCTTTGG), Myrf: (TGGCAACTTCACCTACCACA, GTGGAACCTCTGCAAAAAGC), Mbp: (ACACACGAGAACTACCCATTATGG, AGAAATGGACTACTGGGTTTTCATCT), Plp: (AGCAAAGTCAGCCGCAAAAC, CCAGGGAAGCAAAGGGGG) and GAPDH: (TGACCTCAACTACATGGTCTACA, CTTCCCATTCTCGGCCTTG). The relative expression was calculated by 2−ΔΔct.

### Statistical analysis

Basic descriptive data are presented as means standard errors of means (SEM). For statistical analyses of differences between two groups, paired or unpaired two-tailed Student’s *t* tests were used where appropriate. For experiments involving more than two groups, one-way ANOVA analysis was carried out. Post hoc pairwise comparisons, with Bonferroni correction for multiple comparisons, were conducted where appropriate. An *α*-level of 0.05 was adopted in all instances. All analyses were carried out using SPSS 19 professional software (IBM, USA). Graphs were created using GraphPad Prism (Windows, version 7). None of the samples were excluded from the statistical analysis. Sample sizes referred to the general application of the field and were not statistically predicted. Graphs were created using GraphPad Prism software for Windows, version 5. *p ≤ 0.05, **p ≤ 0.01, ***p ≤ 0.001, ****p ≤ 0.0001, otherwise not significant (n.s.).

## Supplementary information

Supplementary figures and legends

Uncropped blots

## Data Availability

All materials are available upon reasonable request from the corresponding author. All data needed to evaluate the conclusions in the paper are present in the main paper or the Supplementary Materials.
