# Supplementary material for: Palmitoylation regulates myelination by modulating the ZDHHC3-Cadm4 axis in the central nervous system
Source: Signal Transduct Target Ther. 2024 Sep 26;9:254. doi: 10.1038/s41392-024-01971-5 (PMC11427461; doi:10.1038/s41392-024-01971-5)
Supplement: Supplementary file 1 — Supplementary figures and legends [file 41392_2024_1971_MOESM1_ESM.docx]

Supplementary Materials for

Palmitoylation regulates myelination by modulating the ZDHHC3-Cadm4 axis in the central nervous system

Yanli Chang^1,2¶^, Jiangli Zhu^1,3¶^, Xiaopeng Li^1^, Yi Deng^1,2^, Birou Lai^1,2^, Yidan Ma^1^, Jia Tong^2^, Huicong Liu^2^, Juanjuan Li^2^, Chenyu Yang^4^, Qiao Chen^5^, Chengbiao Lu^2^, Yinming Liang^2^, Shiqian Qi^3^, Xiaoning Wang^6*^, Eryan Kong^1,2*^

Correspondence to: eykong2012@163.com, xnwang88@163.com

This PDF file includes:

Materials and Methods

Supplementary Text

Figures. S1 to S15

**Materials and Methods**

*Primary oligodendrocytes Cultures*

Cerebellum isolated from P1 mouse was dissociated with 0.25% trypsin (Gibco, 15090-046) at 37℃ for 20 min, single cells were suspended in DMEM with 10% fetal bovine serum (Gibco) and 100 U/ml penicillin and plated on poly-D-lysine-coated T75 flasks. 7 days later, flasks were shaken at 200rpm/min for 1h at 37℃ to remove microglia; 7 days later, oligodendrocyte precursor cells (OPCs) were collected after shaking 8h at 200rpm/min and maintained in Sato medium (DMEM containing N2 supplement, glutamax, penicillin-streptomycin, 1% fetal calf serum, sodium pyruvate, 0.4mg/ml T3) for about 5-7 days. Medium was replaced every 3–4 days.

*Tissue culture and treatment*

Mouse brains (8 weeks) were rapidly dissected and placed into the ice-cold solution containing 110 mM choline chloride, 2.5 mM KCl, 0.5 mM CaCl_2_, 7 mM MgCl_2_·6H_2_O, 1.3 mM sodium phosphate, 25 mM sodium bicarbonate, and 25 mM glucose. Coronal sections (200μm) were cut on a vibratome (Leica, VT1200S) and cultured in a built-in petri dish(corning3450) with 1.3ml neuro basal medium (Gibco,21103-049) supplement with 5%FBS, 2% B-27, 1% Glumax and 1% penicillin/streptomycin, along with specific drugs or control DMSO at 37 ˚C in a 5% CO_2_ incubator. The following reagents were used for tissue treatments: Dimethyl sulfoxide (DMSO) (Sigma-Aldrich, Cat# D8418), 100 μM Cycloheximide (CHX, MedChemExpress, HY-12320), 50 μM Chloroquine (CHQ, Sigma, C6628), 80 μM dynasore (MedChemExpress, HY-15304), and 800 μM H_2_O_2_. Tissues were collected for analysis after 72 hours of treatment.

*Morris water maze test*

MWM was performed as previously described^1^. Mice at 12 weeks of age were trained for 5 days before the test. They were allowed to swim in a circular pool (120 cm diameter, 50 cm height) filled with water containing white pigment titanium dioxide and divided into four equal quadrants (S1, S2, S3, and S4). During the navigation test, mice were randomly placed in one of the four quadrants and allowed to search for a hidden circular platform (1cm under the water surface) for 1 minute. All mice were then allowed to stay on the platform for 15 seconds at the end of training. In the probe test, the platform was removed and all the mice underwent a 60-second probe trial. The latency to the platform area, speed, and the number of times crossing the platform were recorded using a video tracking system (EthoVision XT15, Noldus Information Technology).

*Open field test*

Open field test was conducted as described previously^1^. An open box measuring 50 cm × 50 cm × 50 cm was used. The floor of the box was divided into 16 equal squares. Mice at 12 weeks of age were placed into the center of the box and allowed to freely explore for 5 minutes. During this time, various parameters including the time spent in the center square, number of entries into the center, total distance traveled, and speed were recorded and analyzed using the EthoVision XT15 system from Noldus Information Technology.

*Rotarod test*

The Rotarod test was used to assess motor coordination. Mice at 12 weeks of age were trained for 5 minutes a day for 3 days on the rotarod machine (YLS-4C Rotary Rod Fatigue Tester, Equipment Station of Shandong Academy of Medical Sciences) at a speed of 20 rpm in a single direction. On the fourth day, the mice were tested for 5 minutes at a speed of 30 rpm, and the time it took for the mice to fall from the rotating rod was recorded.

*Balance beam*

The balance beam test was performed as previously described^1^. The Balance Beam test was performed to evaluate the mice's ability to stay upright and walk across an elevated, narrow beam to reach a safety platform. Mice at 12 weeks of age were tested on a beam that was 100 cm long and 20 mm wide, elevated 50 cm from the floor. They were trained for two consecutive days and then tested on the third day. The latency to cross the beam and the frequency of foot slips during the entire process were recorded using video analysis.

*Patch clamp*

Brains were dissected from Pups (8- to 14-days-old) and placed into an ice-cold slicing solution saturated with 95 % O_2_ and 5% CO_2._ The slicing solution contained 125 mM NaCl, 25 mM NaHCO_3_, 25 mM glucose, 2.5 mM KCl, 1.25 mM NaH_2_PO_4_, 0.1 mM CaCl_2_, and 3 mM MgCl_2_, 0.4 mM ascorbic acid, 3 mM myo-inositol, and 2 mM sodium pyruvate. Coronal brainstem slices (250 μm) containing the medial nucleus of the trapezoid body were cut using a vibratome (Leica, VT1200S). Pipette (3–4 mΩ) were pulled from borosilicate glass (O.D.: 1.5 mm, I.D.: 0.86 mm, Sutter Instrument, USA) and filled with intercellular solution containing (in mM): 125 K-gluconate, 20 KCl, 4 Mg-ATP, 10 Na2-phosphocreatine, 0.3 GTP, 10 HEPES, and 0.5 EGTA (pH 7.2) adjusted with KOH. Postsynaptic whole-cell recordings were obtained on the calyx of Held terminals using a Multiclamp 700B amplifier (Molecular Devices, Sunnyvale, CA, USA), low-pass filtered at 1 kHz, and digitized at 5 kHz using a Digidata 1550B interface (Molecular Devices). The series resistance (< 10 MΩ) was compensated by 98 % (with a lag of 10 μs). Spontaneous excitatory postsynaptic currents (sEPSCs) were recorded at -80 mV under perfusion with a bath solution containing: 125 mM NaCl, 2.5 mM KCl, 1 mM MgCl2, 2 mM CaCl2, 25 mM NaHCO3, 1.25 mM NaH2PO4, 25 mM dextrose, 0.4 mM ascorbic acid, 3 mM myo-inositol, 2 mM sodium pyruvate, 0.01 mM bicuculline. The pH was maintained at 7.4 by bubbling with carbogen (95 % O2, 5 % CO2). To record the evoked EPSCs, stimulation was delivered through a parallel bipolar electrode (30211, FHC, USA) placed at the midline. An A.M.P.I. stimulus isolator (iso-flex, Israel) controlled by the pClamp 10 software (Molecular Devices) drove stimulation pulses (0.1 ms) of varying voltage. Offline analysis was performed using Mini analysis software (version: 6. 0. 3). All patch clamp recordings were conducted at 37℃.

*Single nucleus RNA-seq*

Hippocampus and corpus callosum from the 4-week-old mice were used for single nucleus RNA-seq. The procedure involved quick dissection and freezing of the brain tissue in liquid nitrogen. The frozen tissue was then dounced and microdissected before being placed in nuclei lysis buffer. The homogenized tissue was passed through a cell strainer, and the resulting nuclei were collected by centrifugation. These nuclei were then resuspended in diluted nuclei buffer and subjected to snRNA-seq using the 10x Genomics platform. For data analysis, the quality of the raw reads generated by Illumina sequencers was assessed using the fastp tool. The number of filled cells was determined for each sample: WT (9970 cells), Cadm4-KI (13756 cells), and ZDHHC3-KO (11684 cells). Gene-barcode matrices were generated using the Cellranger software. Raw reads were demultiplexed and mapped to the reference genome using UMI-tools. All subsequent single-cell analyses were performed using the Seurat package. Cells with less than 200 genes or high levels of red blood cell-related genes and mitochondrial genes were filtered out. The filtered gene expression matrix was log normalized and scaled. Highly variable genes were selected, and canonical correlation analysis (CCA) was applied to find anchors for integrated data. Clustering and visualization were performed using methods such as RunUMAP, RunTSNE, FindNeighbors, and FindClusters with PCA reduction and a resolution of 0.6. Marker genes for each cluster were identified using FindAllMarkers, and only marker genes with a padj (Benjamini-Hochberg adjusted p-value) of less than 0.05 were retained. Violin plots and t-SNE plots were created for the top four marker genes within each cluster. Gene Ontology (GO) enrichment analysis of marker genes was conducted using the clusterProfiler package, correcting for gene length bias. GO terms with a corrected p-value below 0.05 were considered significantly enriched by marker genes. KEGG pathway enrichment analysis of marker genes was performed using the clusterProfiler package. CytoTRACE analysis was carried out with default parameters to evaluate the differentiation states based on gene expression information obtained from snRNA-seq data. By comparing the expression levels of each gene in a single cell to the expression levels in the entire population of cells, the relative positions of cells during development can be determined.

*Lysolecithin induced demyelination*

Lysolecithin (LPC) induced demyelination was conducted following previously described methods^2^. Female mice (8 weeks) were deeply anaesthetized using 1% pentobarbital sodium and then injected with 1% lysolecithinsolution (lysolecithin L4129, Sigma) dissolved in phosphate-buffered saline into the corpus callosum. The injection coorinates were 1 mm lateral to bregma, 1.49 mm anterior to bregma, and 2.1 mm deep to the cortical surface using a stereotaxic apparatus (Reaworld, D01996-003). A total of 1.5 μL LPC was injected at a rate of 50 nL/min using a pump (Harvard, 704507). Mice were euthanized on day 7 and day 21 post-injection to collect brains for Transparency and Immunohistofluorescence, RAC assay, and qRT-PCR analysis.

**
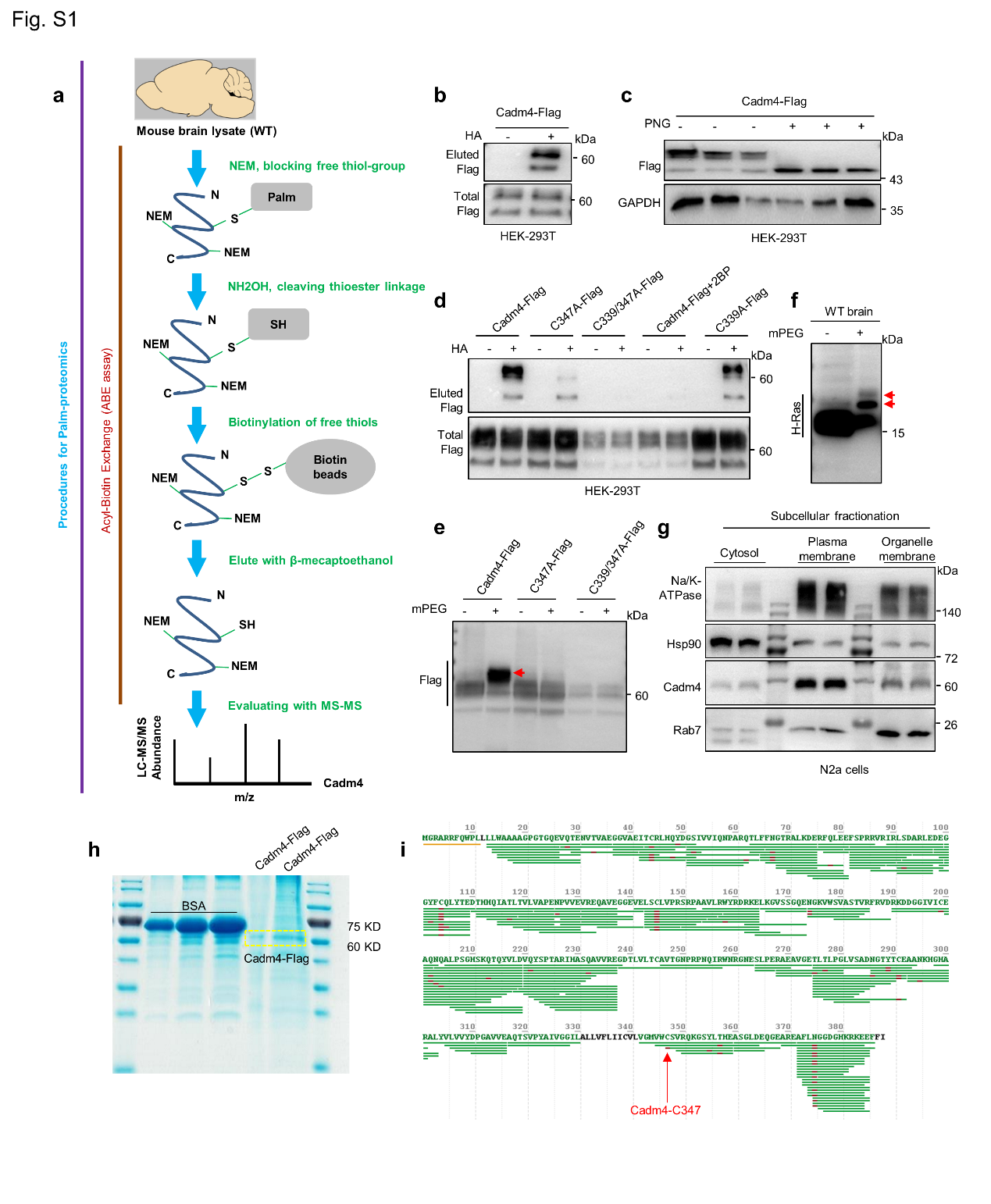
Fig. S1. Identification of Cadm4 as palmitoylated protein. a**, Diagram to show the procedures of palm-proteomics, where Cadm4 was initially identified. **b**, Cadm4 expressed in HEK-293T cells was analyzed for palmitoylation by Acyl-RAC assay. **c**, HEK-293T cells expressing Cadm4 was incubated with or without PNG (an inhibitor of protein glycosylation) for WB analysis. **d**, Cadm4 or its mutants (C347A and C339A/C347A) were expressed in HEK-293T cells and subjected for Acyl-RAC assay. **e**, HEK cells expressing Cadm4 or its mutants (C347A and C339A/C347A) were processed for mPEG-labeling assay. The migrated band is pointed by the red arrow. **f**, WT brain lysate was processed with mPEG-labeling assay for detecting H-Ras palmitoylation. The mPEG labeling causes the band shift, pointed with red arrows. **g**, Subcellular fractions were prepared from WT N2a cells, HSP-90 is a marker for cytosol proteins, Na/K ATPase is a marker for plasma membrane proteins. **h**, Purified Cadm4 stained with coomassie brilliant blue. **i**, Purified Cadm4 was probed by mass spectrometry, and the coverage of matched peptides was demonstrated.


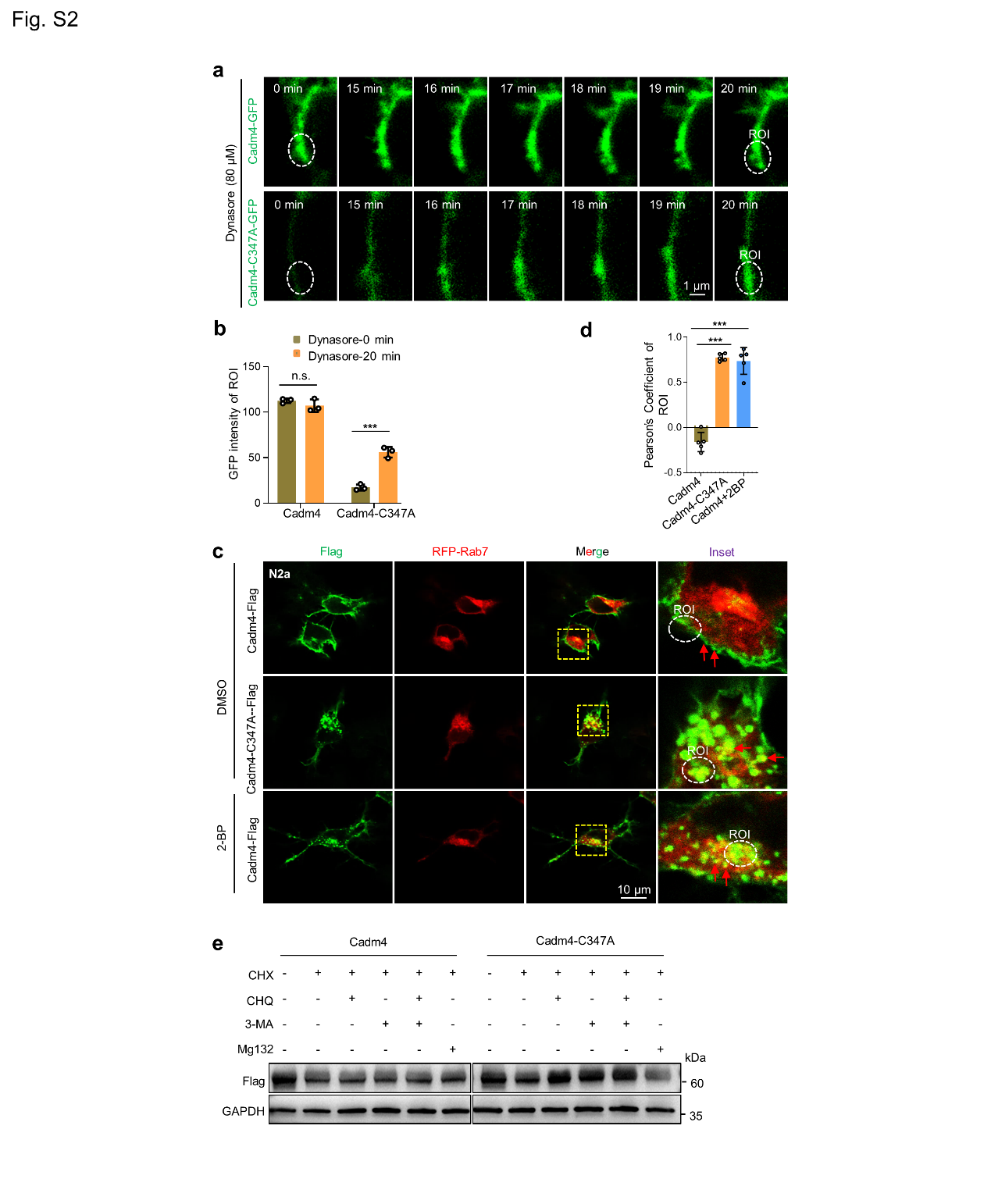


**Fig. S2. Dynasore inhibits Cadm4 internalization induced by attenuated level of Cadm4 palmitoylation. a**, N2a cells expressing Cadm4-GFP/Cadm4-C347A-GFP were treated with Dynasore for time-lapse imaging (**a**), GFP intensity was quantified in ROI at different timepoints (**b**, 2-tailed t-test, n=3). **c-d**, Cadm4-GFP/Cadm4-C347A-GFP was expressed in N2a cells, treated with DMSO or 2-BP (50 µM) and fixed for immunofluorescence imaging, RFP-Rab7 is a marker for late endosome (**c**), the colocalization of Cadm4/Rab7 was quantified (**d**). one-way ANOVA followed by Bonferroni post hoc test, n=5, Cadm4 and Cadm4-C347A, *** p≤0.001; Cadm4 and Cadm4+2-BP, ****P*≤0.001. Data are represented as mean±SEM.


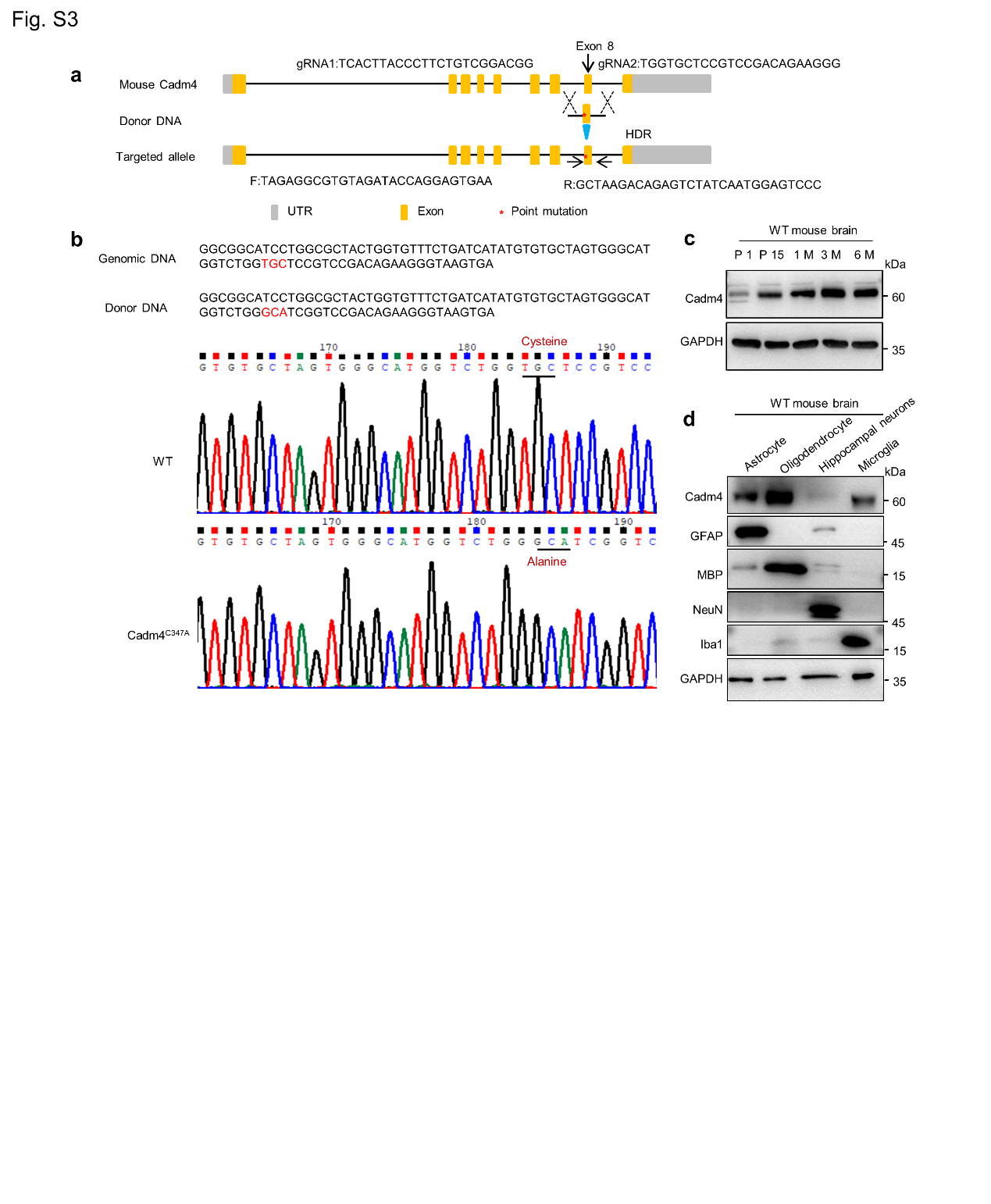


**Fig. S3. Generation of Cadm4-C347A point mutation mouse. a**, Targeting scheme of the point mutation (C347A) in the 8th exon of mouse Cadm4, the point mutation was introduced into mouse fertilized eggs using Crispr/Cas9 with the HDR donor DNA. Yellow box indicates exons, gray box indicates UTRs. Red star indicates a TGC→GCA mutation in the 8th exon of mouse Cadm4; **b**, The sequences of WT genomic DNA and donor DNA. TGC to GCA (red) was designed to change the amino acid Cys to Ala; and representative sequencing result of Cadm4-C347A point mutation mouse. The mutation TGC to GCA was underlined and the changed amino acid Cys to Ala was in dark-red. **c**, The protein level of Cadm4 was analyzed at different developmental stages of WT mouse brain. **d**, Varied cell types were isolated and cultured from P0 WT mouse brain, and Cadm4 level was analyzed by WB.

**
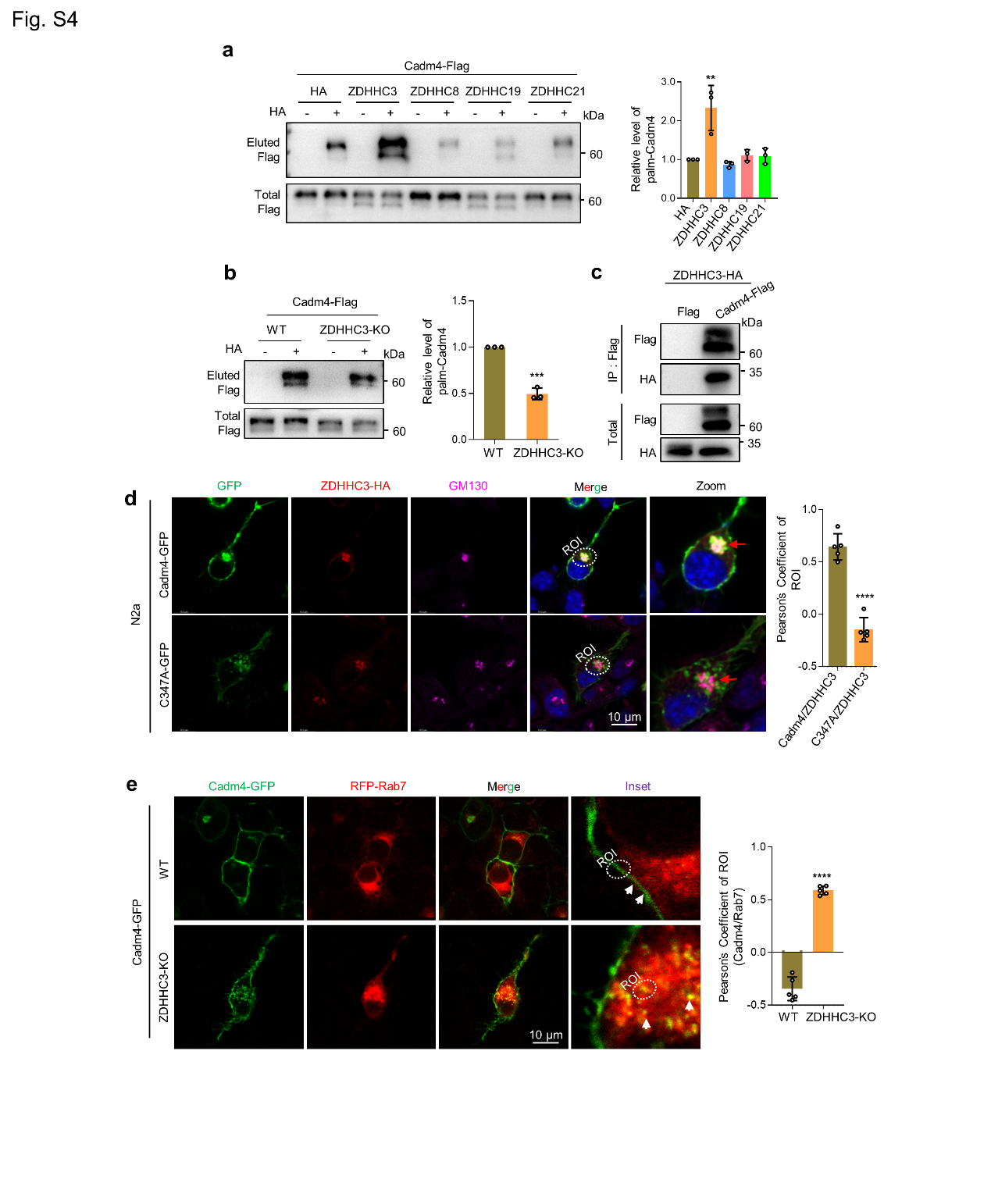
**

**Fig. S4. The identification of ZDHHC3 that catalyzes Cadm4 palmitoylation. a**, DHHCs were coexpressed with Cadm4 in N2a cells for evaluating the level of palm-Cadm4 by Acyl-RAC, and quantified (one-way ANOVA followed by Bonferroni post hoc test, n=3, ***P*<0.01). **b**, Cadm4-Flag was expressed in WT/ZDHHC3-KO N2a cells for the evaluation of palm-Cadm4, and quantified (2-tailed t-test, n=3, ****P*≤0.001). **c**, Lysate of N2a cells expressing Cadm4-Flag/ZDHHC3-HA was collected, Flag-conjugated agarose beads were used for IP analysis. **d**, N2a cells expressing Cadm4-GFP/Cadm4-C347A-GFP and ZDHHC3-HA were fixed for immunofluorescence imaging, the colocalization of Cadm4/ZDHHC3 is quantified (2-tailed t-test, n=5, *****P*≤0.0001). GM130 is a marker of Golgi. **e**, Cadm4-GFP was expressed in WT/ZDHHC3-KO N2a cells for colocalization analysis (2-tailed t-test, n=5, *****P*≤0.0001), RFP-Rab7 is a marker for late endosome. Data are represented as mean±SEM.

**
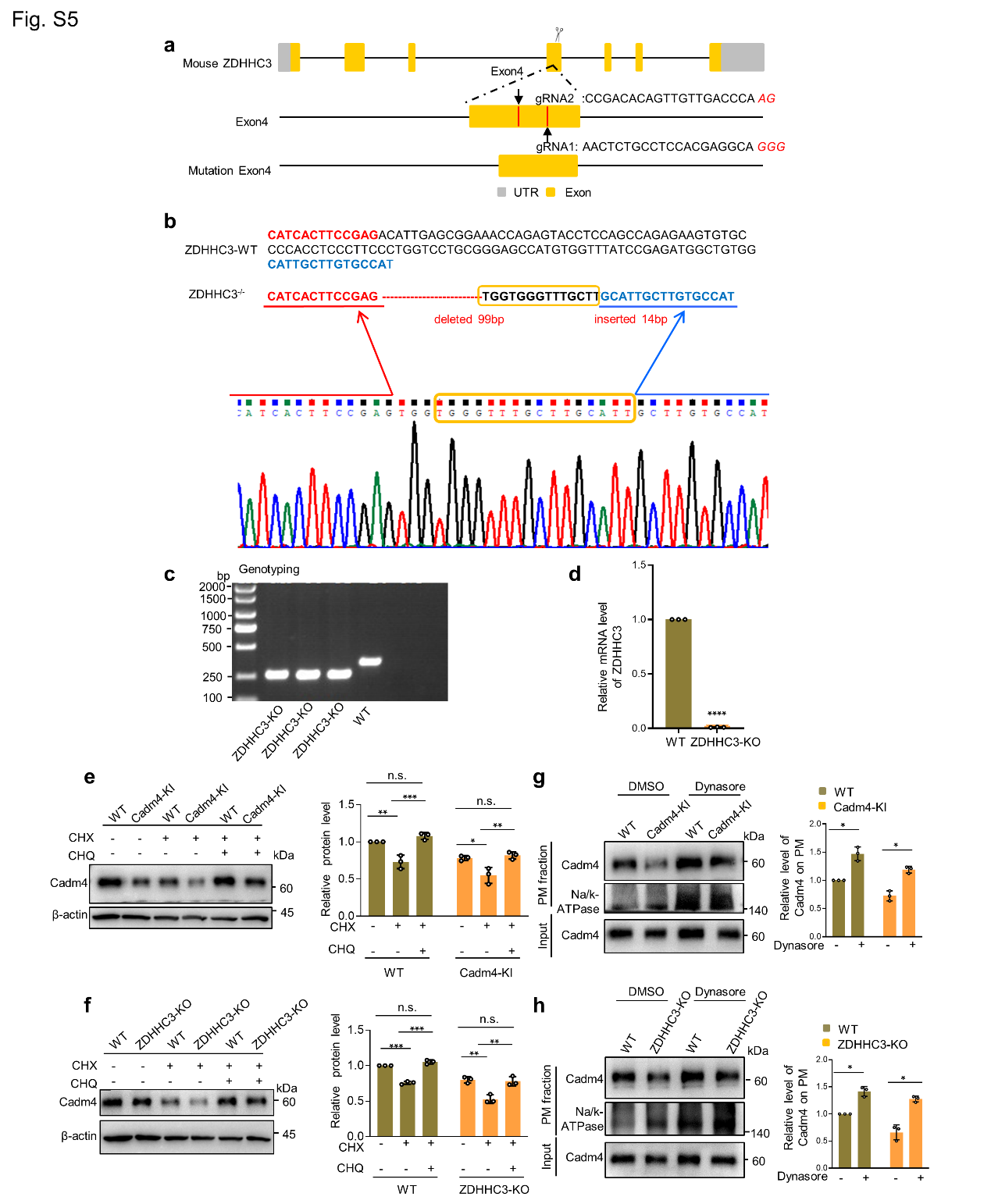
**

**Fig. S5. Generation of ZDHHC3-KO mouse. a**, Targeting scheme of the truncation in the 4th exon of mouse ZDHHC3, where the enzyme activity center locates. The deletion mutation was introduced into mouse fertilized eggs (C57/B6 background) using Crispr/Cas9 with two gRNA. Red box indicates exons, gray box indicates UTRs. Red lines indicate two cut sites in the 4th exon of mouse ZDHHC3. **b**, The sequences of WT genomic DNA and mutation DNA. 99bp were deleted from the mutation DNA, but inserted 14 bp, together it caused frameshift and an earlier stopcodon. A representative sequencing result of ZDHHC3-KO mouse. The insertion was outlined. The evidence at the protein level was not shown because the commercially available ZDHHC3-antibody lacks specificity. **c**, The genotyping of ZDHHC3-KO mouse by PCR. **d**, qRT-PCR for ZDHHC3-KO mouse. **e** and **f**, Brain sections from WT and Cadm4-KI (**e**), and ZDHHC3-KO (**f**) were incubated with or without CHX, CHQ and subjected for WB analysis, and quantified. **g** and **h**, Brain sections from WT and Cadm4-KI (**g**), and ZDHHC3-KO (**h**) were incubated with or without Dynasore and subjected for WB analysis, and quantified. One-way ANOVA followed by Bonferroni, *** P <0.001, ** P <0.01, *P <0.05. Data are represented as mean±SEM.

**
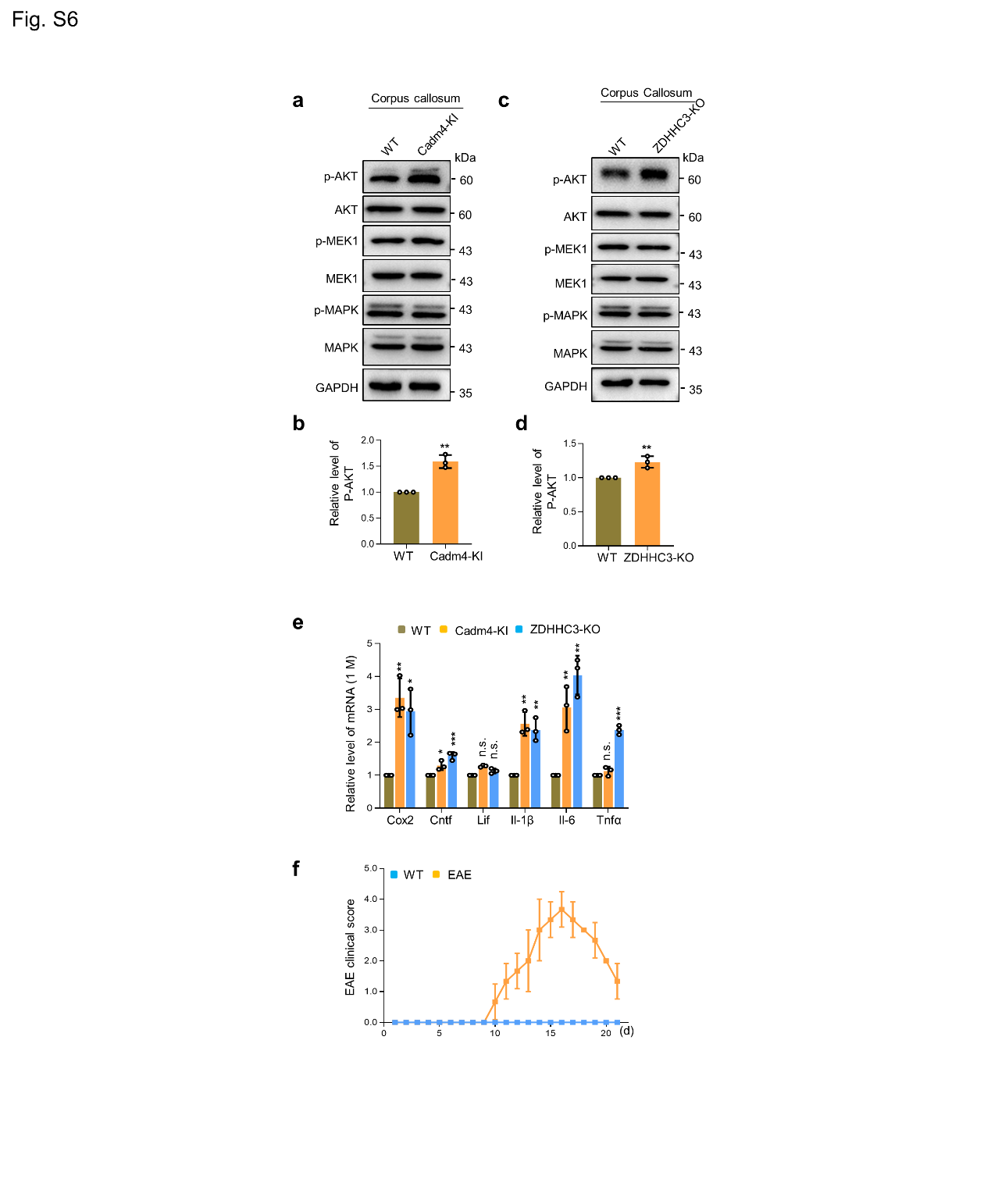
**

**Fig. S6. Phospho-AKT is upregulated in the corpus callosum of Cadm4-KI and ZDHHC3-KO mice. a-b**, Lysates (prepared with phosphatase inhibitors) from either the corpus callosum of Cadm4-KI (**a**) or ZDHHC3-KO (**c**) mouse were analyzed for the levels of various proteins related to the classical signaling cascades downstream of PI3K, and the relative level of p-AKT/total AKT was quantified (**b, d**). 2-tailed t-test, n=3, ***P*≤0.01. **e**, Relative mRNA level of neuroinflammation markers was evaluated by Real-time PCR in different genotypes (4-weeks old). One-way ANOVA followed by Bonferroni post hoc test, n=3. **f**, The clinical score of EAE disease model was evaluated. Data are represented as mean±SEM.


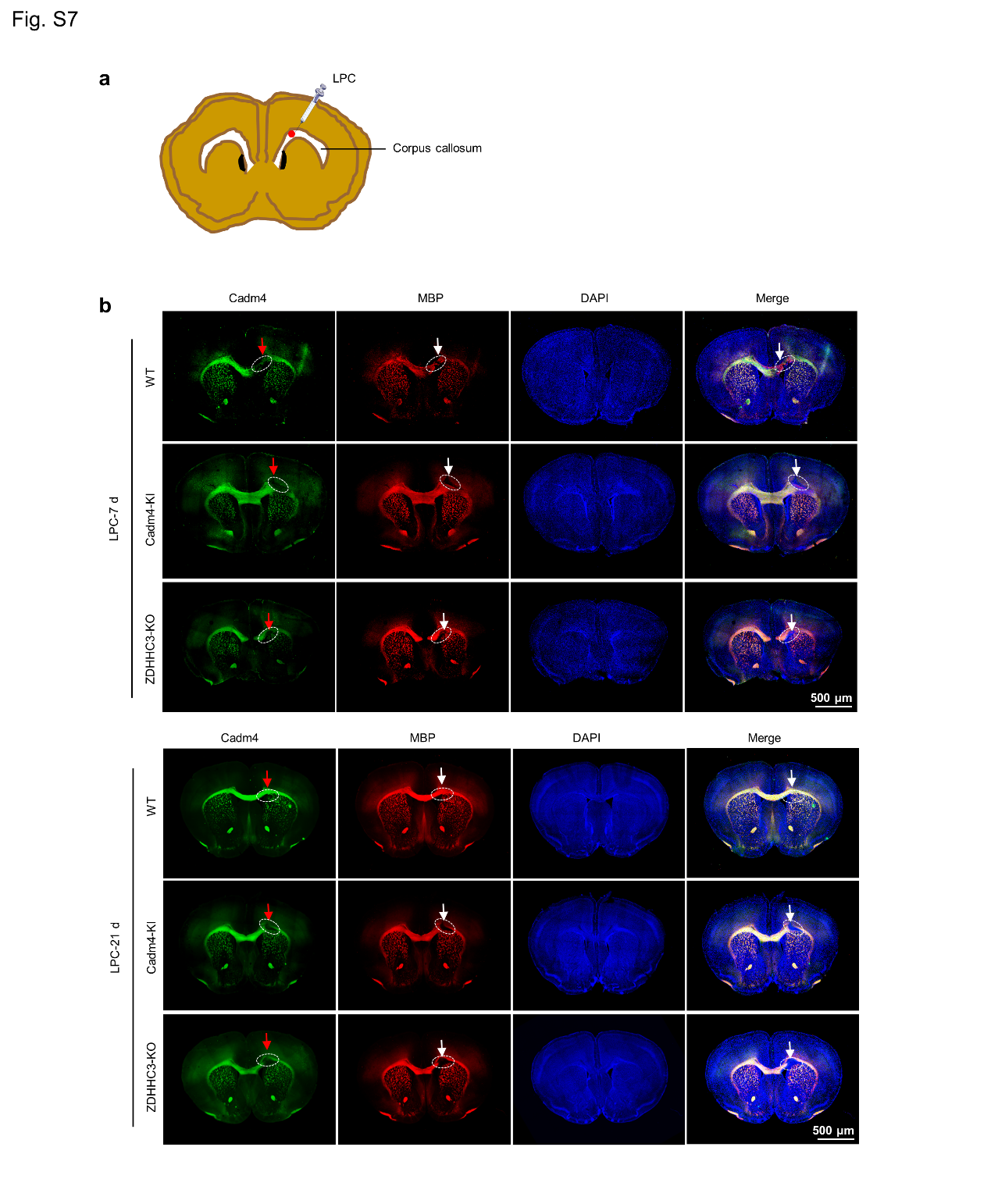


**Fig. S7. Inhibiting Cadm4 palmitoylation suppresses remyelination in LPC demyelination model. a**, Schematic diagram to illustrate the induction of LPC in Corpus callosum. **b**, Mice brains from different genotypes at 7- and 21-days post LPC injection were processed for transparency using X-Clarity and stained for Cadm4, MBP, and DAPI. Red and white arrows were used to indicate the location of demyelination.

**
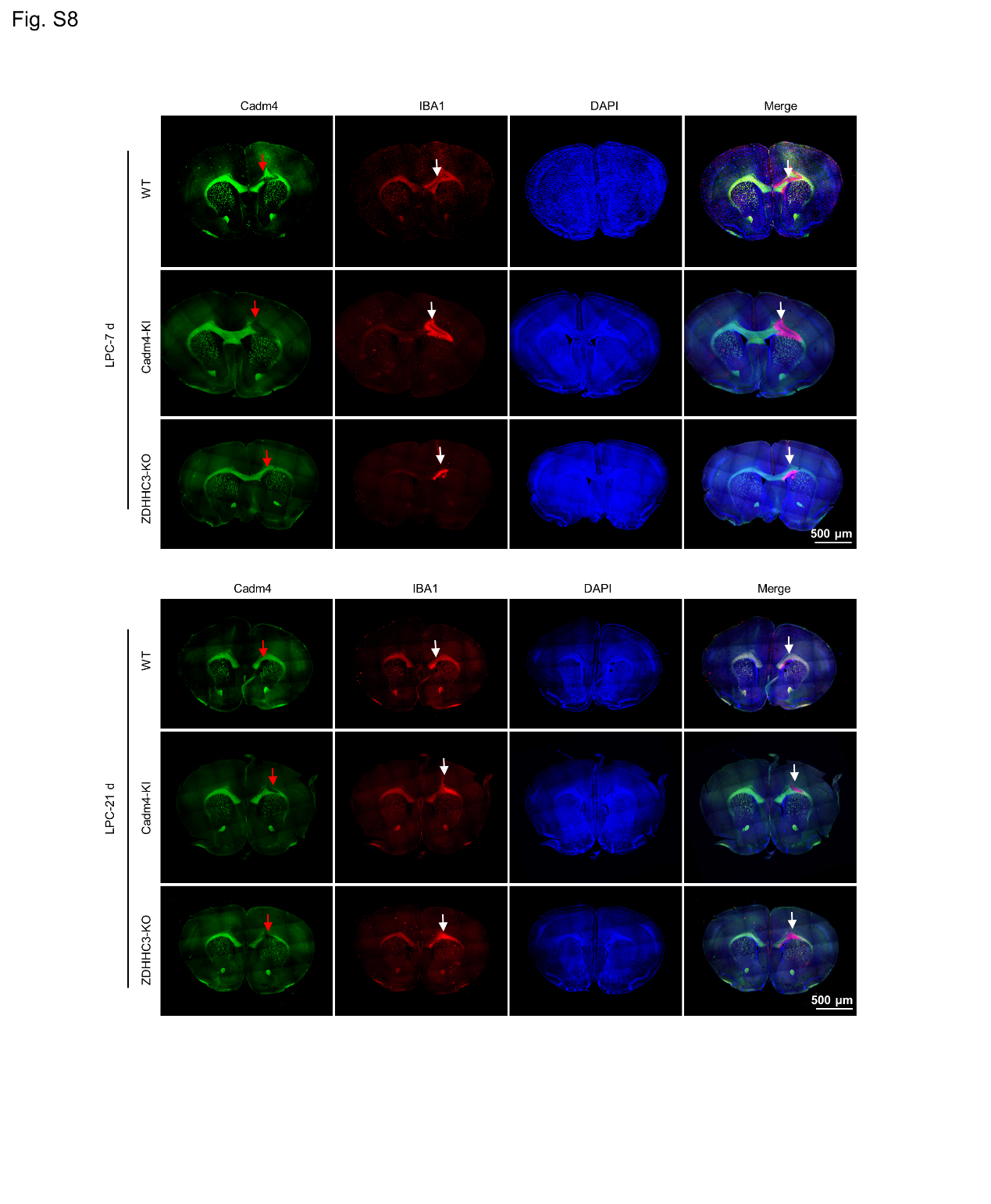
**

**Fig. S8. Inhibiting Cadm4 palmitoylation results in prolonged activation of microglia in LPC demyelination model.** Mice brains from different genotypes at 7- and 21-days post LPC injection were processed for transparency using X-Clarity and stained for Cadm4, Iba1, and DAPI. Red and white arrows were used to indicate the location of demyelination.

**
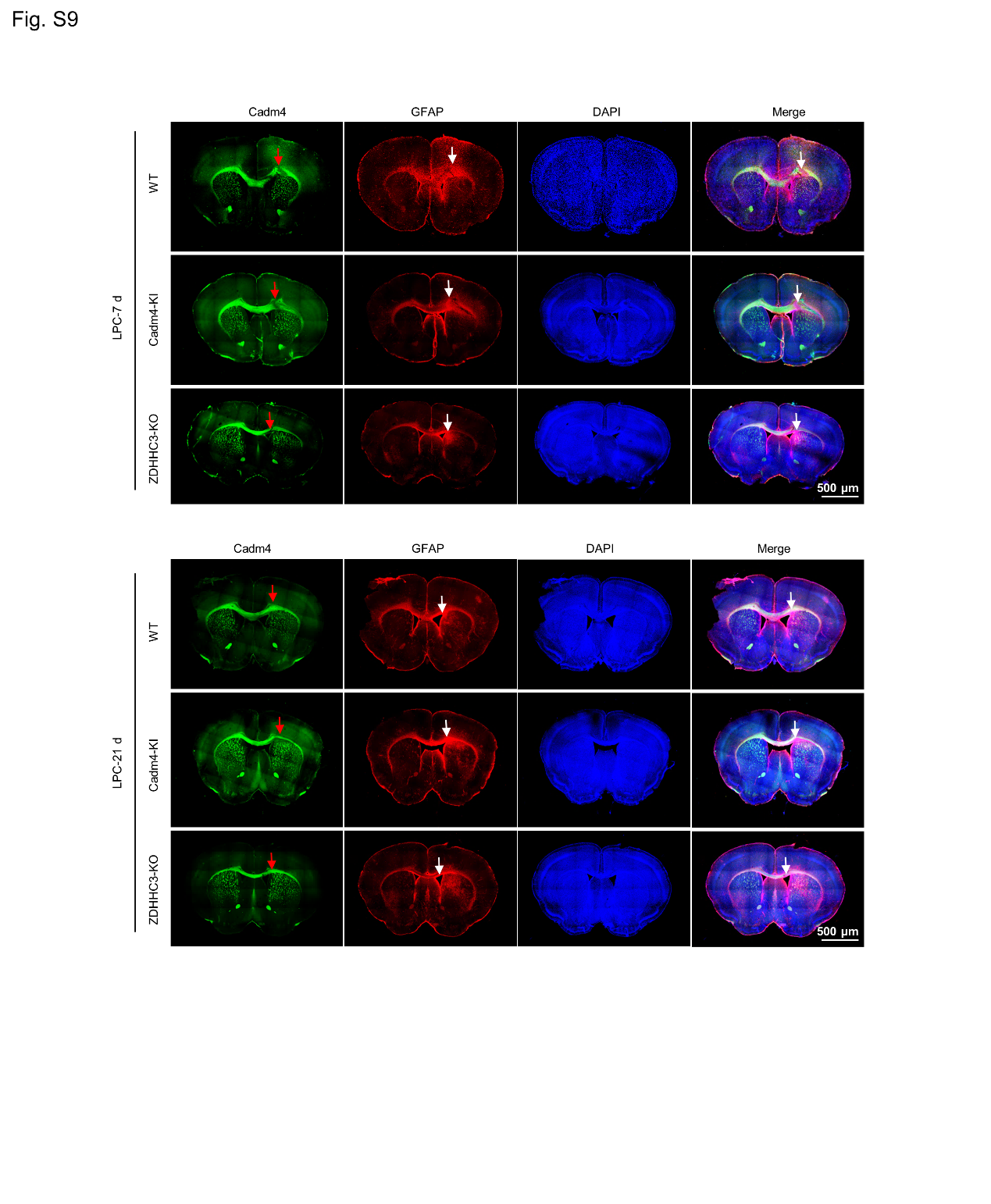
**

**Fig. S9. Inhibiting Cadm4 palmitoylation leads to prolonged activation of astrocyte in LPC demyelination model.** Mice brains from different genotypes at 7- and 21-days post LPC injection were processed for transparency using X-Clarity and stained for Cadm4, GFAP, and DAPI. Red and white arrows were used to indicate the location of demyelination.

**
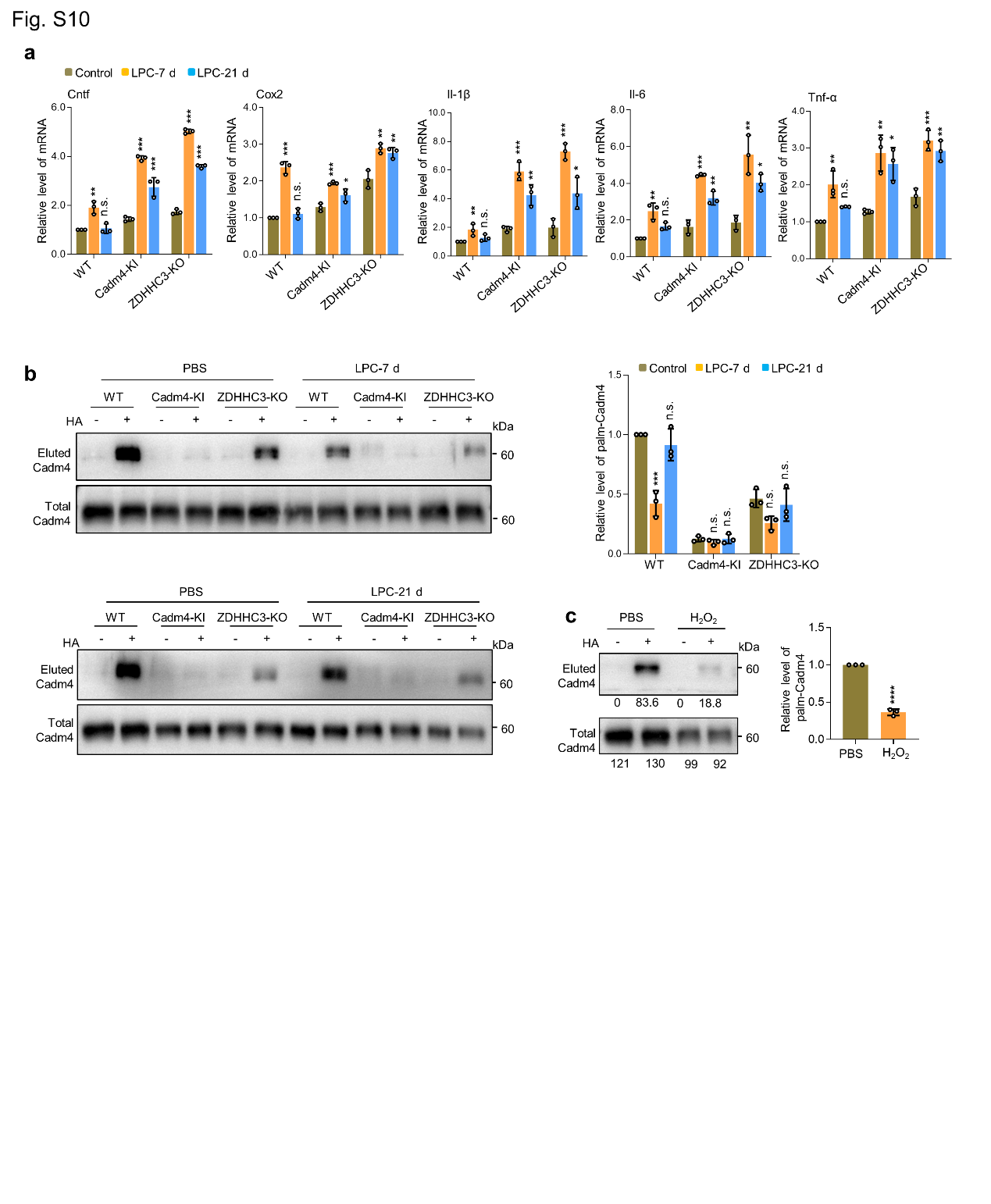
**

**Fig. S10. Inhibiting Cadm4 palmitoylation hinders the recovery process in LPC demyelination model. a**, Relative mRNA level of neuroinflammation markers was evaluated at different timepoints by Real-time PCR in different genotypes. One-way ANOVA followed by Bonferroni post hoc test, n=3. **b**, The level of palm-Cadm4 was evaluated in different genotypes in LPC demyelination model, and quantified. One-way ANOVA followed by Bonferroni post hoc test, n=3. **c**, The level of palm-Cadm4 was evaluated in brain sections treated with H_2_O_2._ Data are represented as mean±SEM.

**
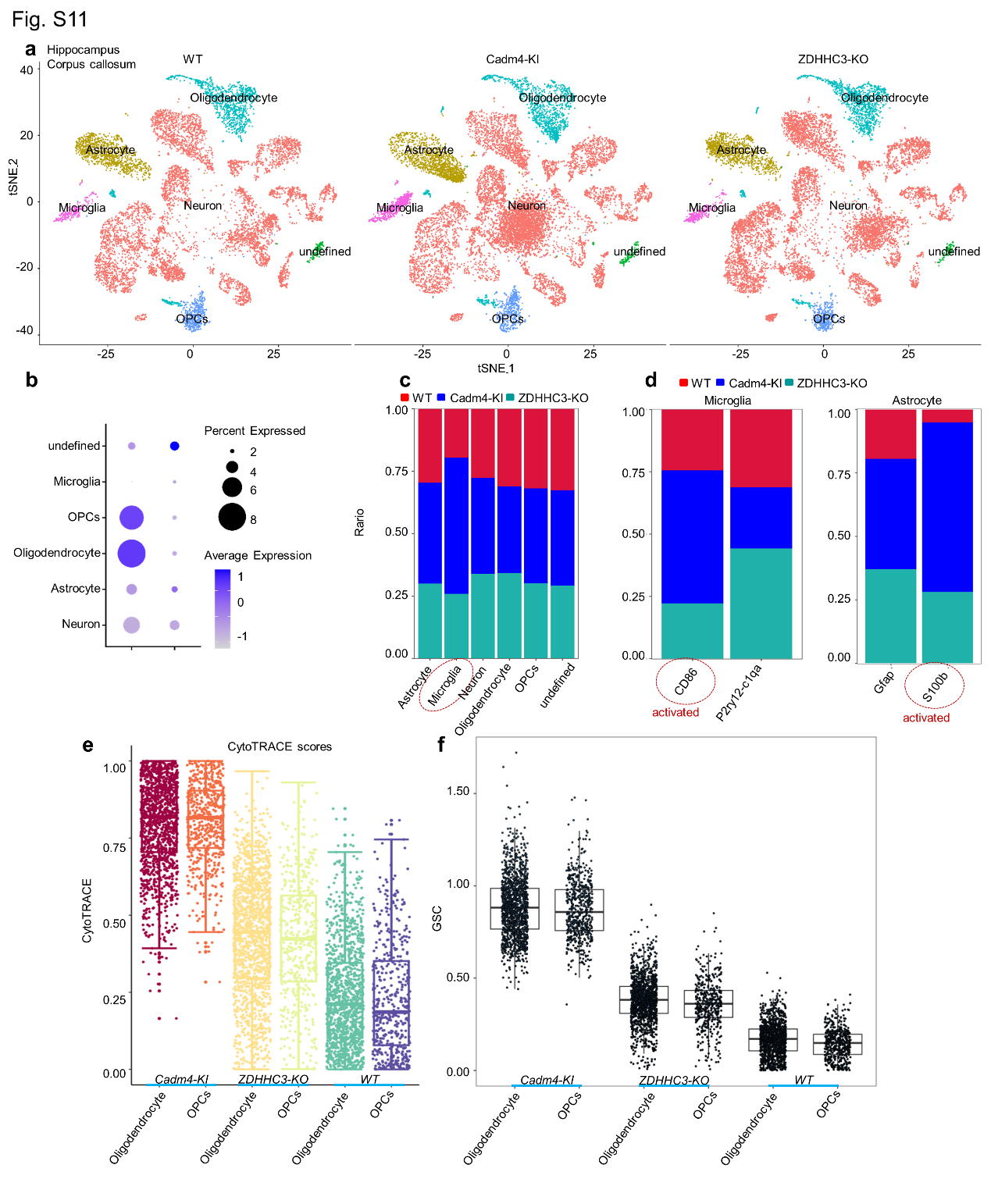
Fig. S11. Single-nucleus sequencing analysis of mice brains with different genotypes. a**, t-SNE plot showing 24 distinguished clusters, Neuron, Oligodendrocyte, OPC, Microglia and Astrocyte with cell-type identities as determined by expression of specific markers: Neuron (Syt1, Rbfox1, Rbfox3), Oligodendrocyte (Plp1, Mbp, Cldn11), OPC (Vcan, Olig1, Cspg4), Microglia (Cx3cr1, C1qb, C1qa) and Astrocyte (Aqp4, Slc1a3, Gfap, Slc1a2). **b**, Expression profiles of Cadm4 and ZDHHC3 were illustrated in different cell clusters. **c**, The percentage of different cell types were compared in different genotypes. **d**, The percentage of CD86-positve (activating state) and p2ry12-C1qa-positive (resting state) cell clusters in microglia population, and S100b-positive (activating state) and GFAP-positive in astrocyte population were compared in different genotypes. **e**, CytoTRACE analysis of OPCs and Oligodendrocytes in different genotypes. **f**, GSC analysis of OPCs and Oligodendrocytes in different genotypes.

**
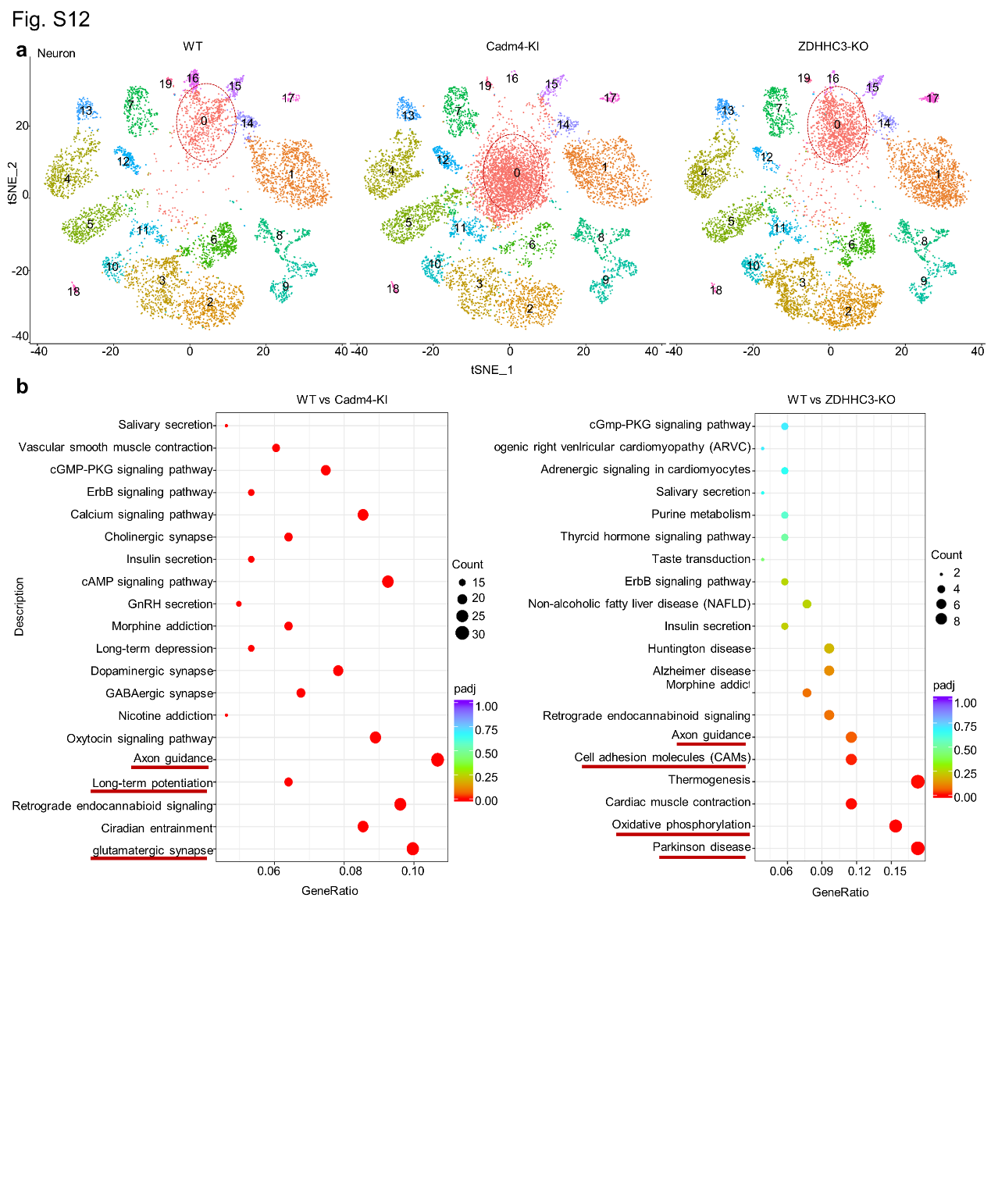
**

**Fig. S12. Single-nucleus sequencing analysis of neuron clusters with different genotypes. a**, t-SNE plot showing 19 distinguished sub-clusters in neuron population. **b**, The circulated sub-cluster of neuron population was selected for further KEGG analysis between different genotypes.

**
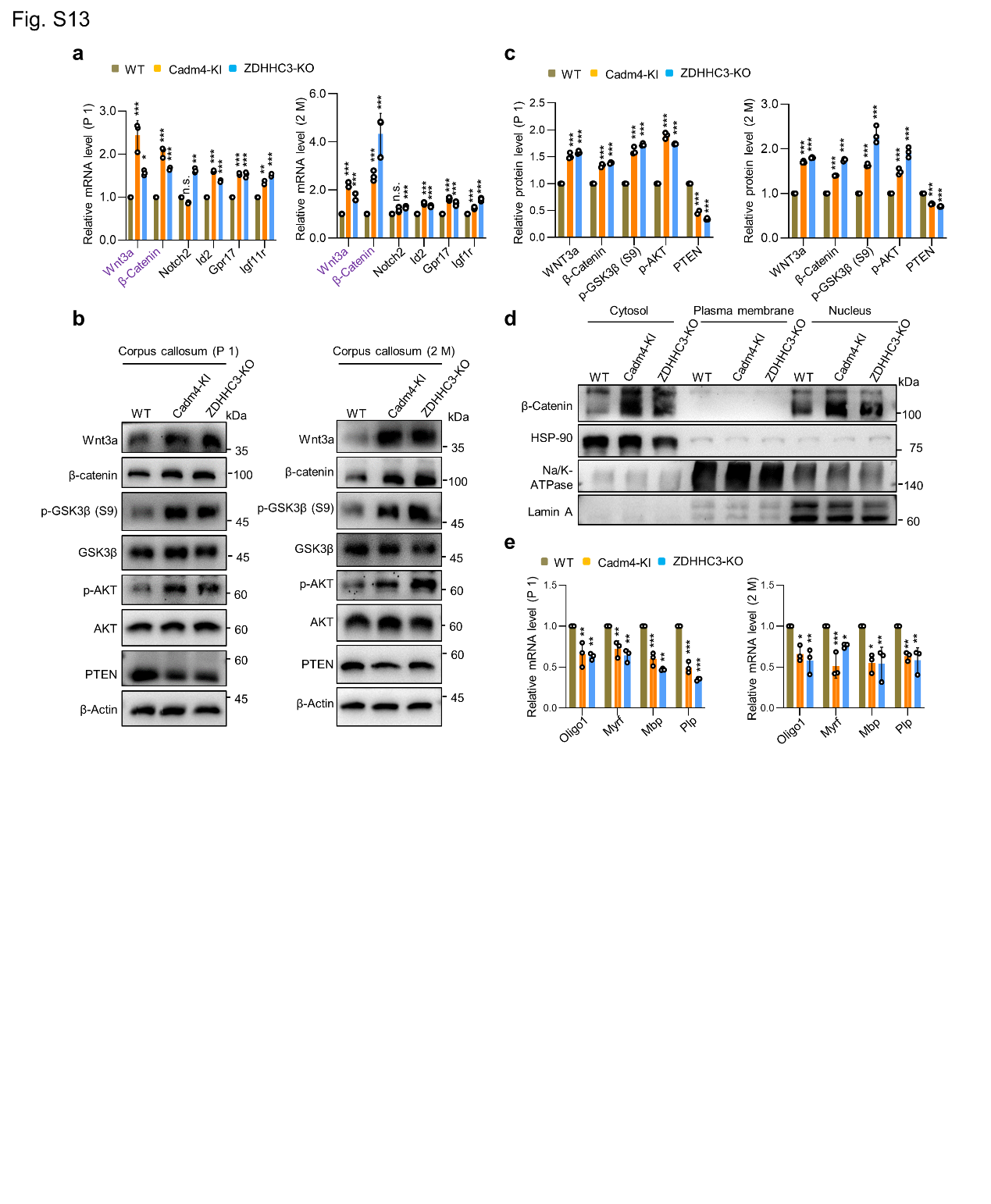
**

**Fig. S13. Modulation of the WNT-β-Catenin signaling pathway in the corpus callosum of Cadm4-KI and ZDHHC3-KO mice.** **a**, The relative mRNA levels of key molecules in various signaling pathways (NOTCH/WNT-β-Catenin/IGF/GPR17-ID2) were evaluated at different time points by Real-time PCR in the corpus callosum of different genotypes. One-way ANOVA followed by Bonferroni post hoc test, n=3. **b-c**, The corpus callosum was isolated from different genotypes and subjected to evaluation of protein levels by western blotting, which were subsequently quantified. **d**, The corpus callosum collected from different genotypes underwent subcellular fractionations. **e**, The relative mRNA levels of key lineage markers of OPC or oligodendrocyte were evaluated at different time points by Real-time PCR in the corpus callosum of different genotypes. One-way ANOVA followed by Bonferroni post hoc test, n=3. Data are represented as mean±SEM.

**
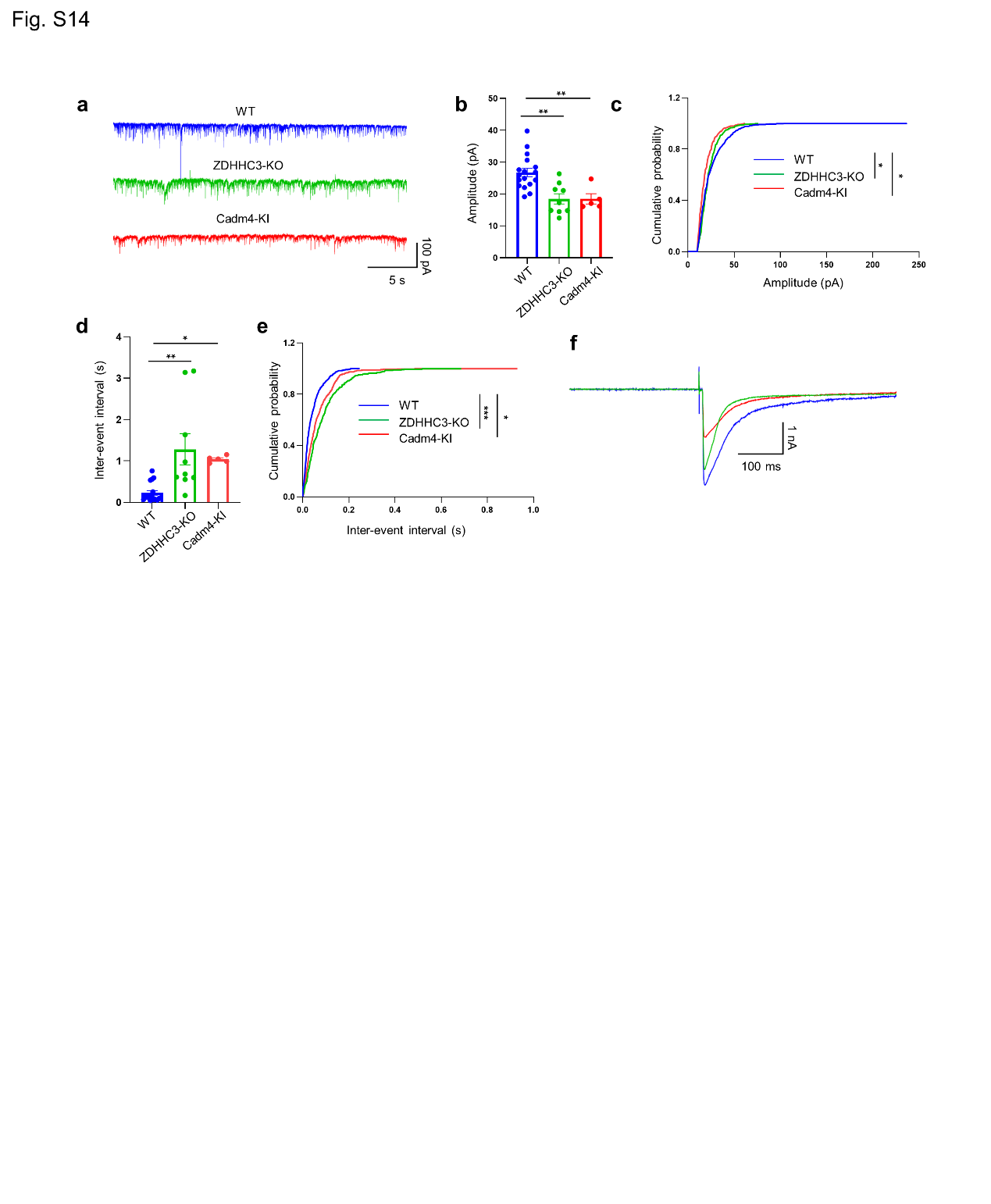
**

**Fig. S14. Inhibiting Cadm4 palmitoylation weakens neuronal conduction. a**, Sample traces of sEPSCs recorded at postsynaptic principal cells from different genotypes. **b**, Amplitude of sEPSCs from different genotypes were quantified. WT (n=17 cells from 12 slices); ZDHHC3-KO (n= 9 cells from 6 slices); Cadm4-KI (n= 5 cells from 5 slices). One-way ANOVA followed by Bonferroni, *** P <0.001, ** P <0.001, *P <0.05. **c**, Cumulative probability curve of amplitude. Kolmogorov-Smirnov Test, * P <0.05. **d**, Inter-event intervals of sEPSCs from different genotypes. One-way ANOVA followed by Bonferroni, *** P <0.001, ** P <0.001, *P <0.05. **e**, Cumulative probability curve of Inter-event intervals. Kolmogorov-Smirnov Test, *** P <0.001; *P <0.05. **f**, Sample traces of evoked EPSCs recorded at postsynaptic principal cells. Data are represented as mean±SEM.

**
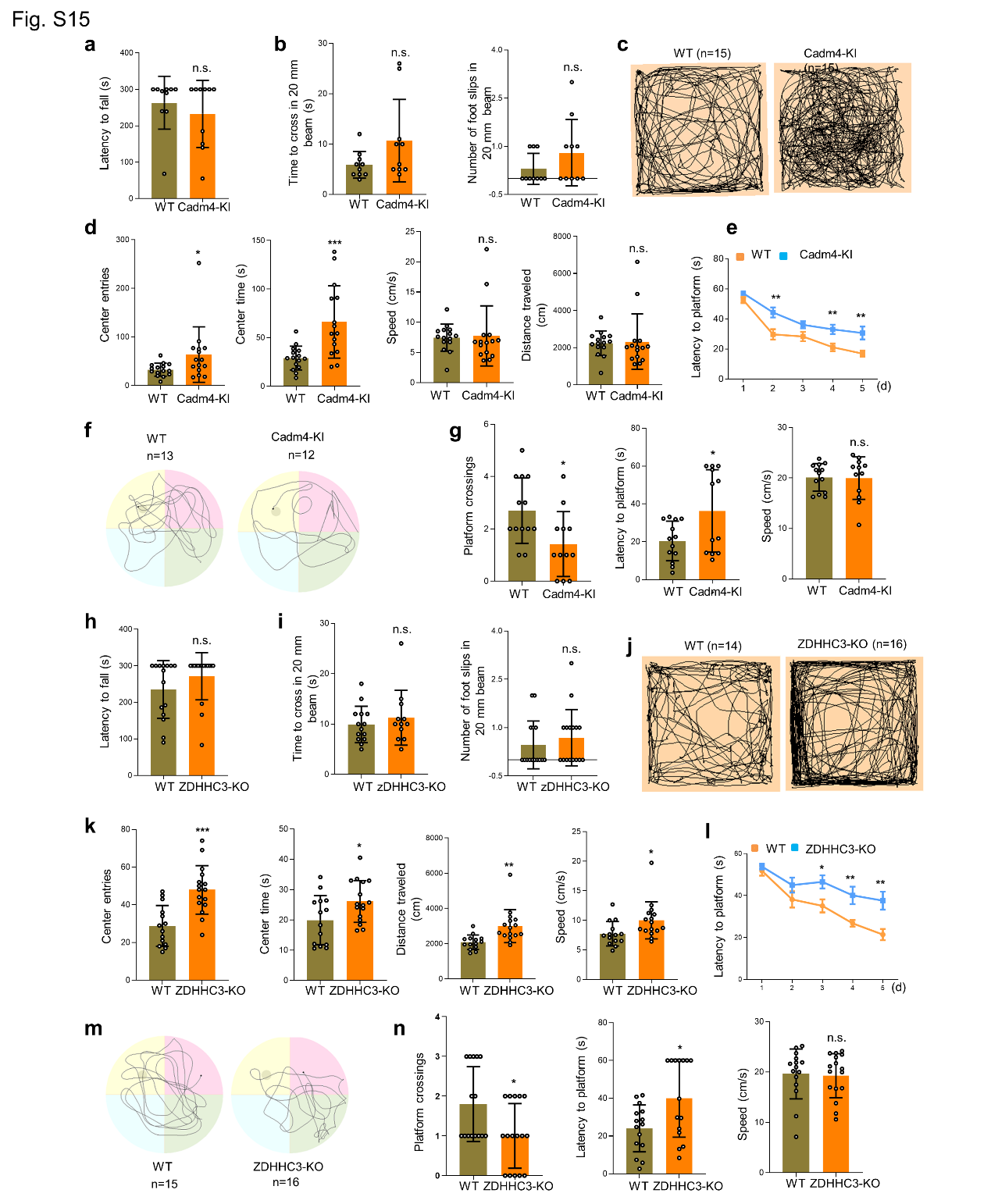
**

**Fig. S15. Inhibiting Cadm4 palmitoylation leads to altered behavior features in mice.** **a-g**, WT (n=10-15) and Cadm4-KI (n=10-15) mice were tested with Rotarod test (**a**), balance beam test (**b**), Open field test (**c-d**), Morris water-maze test (**e-g**), and corresponding parameters were quantified. **h-n**, WT (n=14-15) and ZDHHC3-KO (n=12-16) mice were tested with Rotarod test (**h**), balance beam test (**i**), Open field test (**j-k**), Morris water-maze test (**l-n**), and corresponding parameters were quantified. two-tailed t-test, *P < 0.05, **P ≤ 0.01 and ***P ≤ 0.001. Data are represented as mean±SEM.

**References**

1 Liu, H. *et al.* Palmitoylated Sept8-204 modulates learning and anxiety by regulating filopodia arborization and actin dynamics. *Sci Signal* **16**, eadi8645 (2023).

2 Duncan, G. J. *et al.* Myelin regulatory factor drives remyelination in multiple sclerosis. *Acta Neuropathol* **134**, 403-422 (2017).
