# Supplementary material for: Palmitoylation regulates myelination by modulating the ZDHHC3-Cadm4 axis in the central nervous system
Source: Signal Transduct Target Ther. 2024 Sep 26;9:254. doi: 10.1038/s41392-024-01971-5 (PMC11427461; doi:10.1038/s41392-024-01971-5)
Supplement: Supplementary file 2 — Uncropped blots [file 41392_2024_1971_MOESM2_ESM.docx]

Supplementary Materials for

Palmitoylation regulates myelination by modulating the ZDHHC3-Cadm4 axis in the central nervous system

Yanli Chang^1,2¶^, Jiangli Zhu^1,3¶^, Xiaopeng Li^1^, Yi Deng^1,2^, Birou Lai^1,2^, Yidan Ma^1^, Jia Tong^2^, Huicong Liu^2^, Juanjuan Li^2^, Chenyu Yang^4^, Qiao Chen^5^, Chengbiao Lu^2^, Yinming Liang^2^, Shiqian Qi^3^, Xiaoning Wang^6*^, Eryan Kong^1,2*^

Correspondence to: eykong2012@163.com, xnwang88@163.com

This PDF file includes:

Uncropped blots

Figures. S16 to S22

**
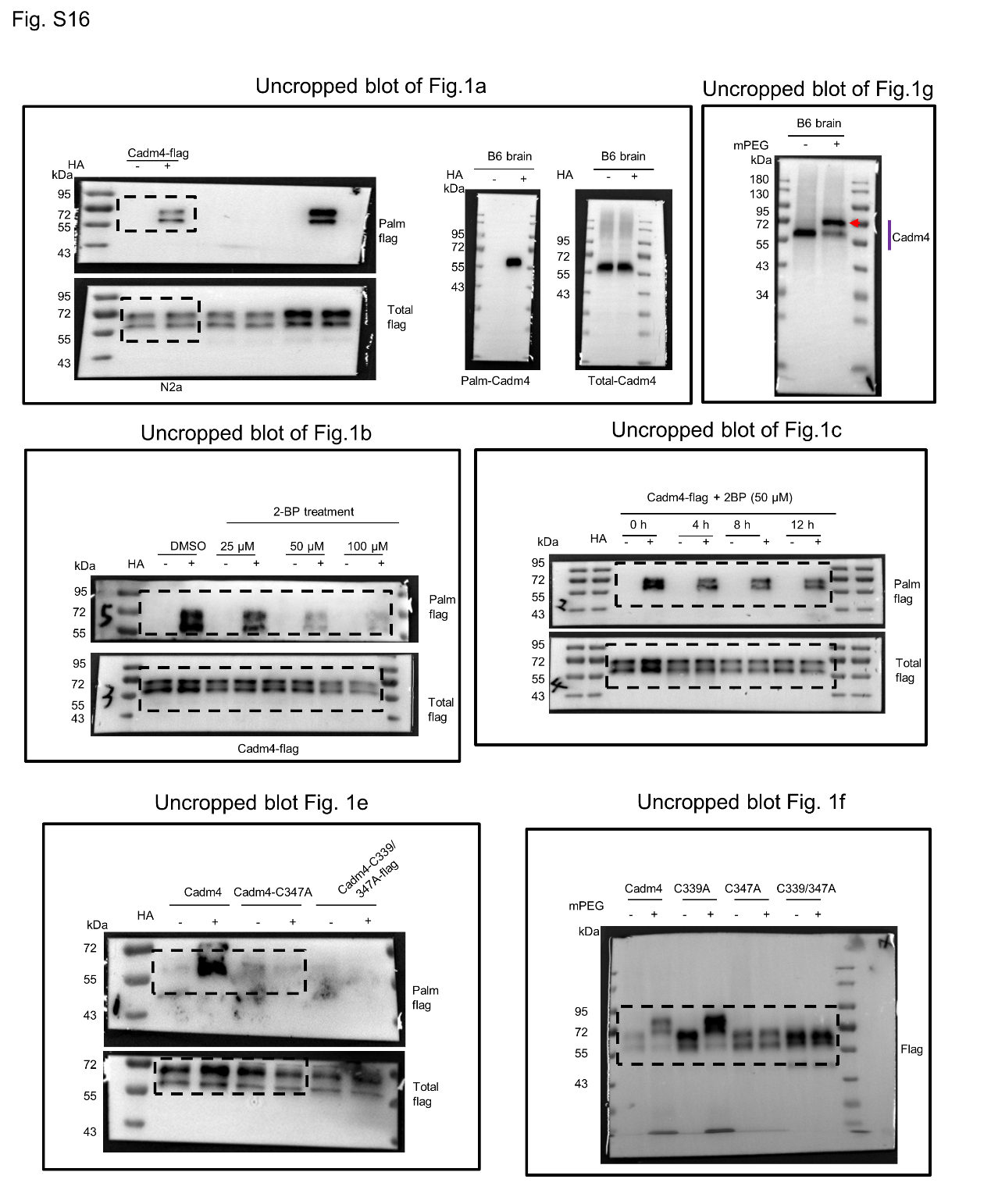
**

**Fig. S16. Uncropped blots for Fig. 1.**

**
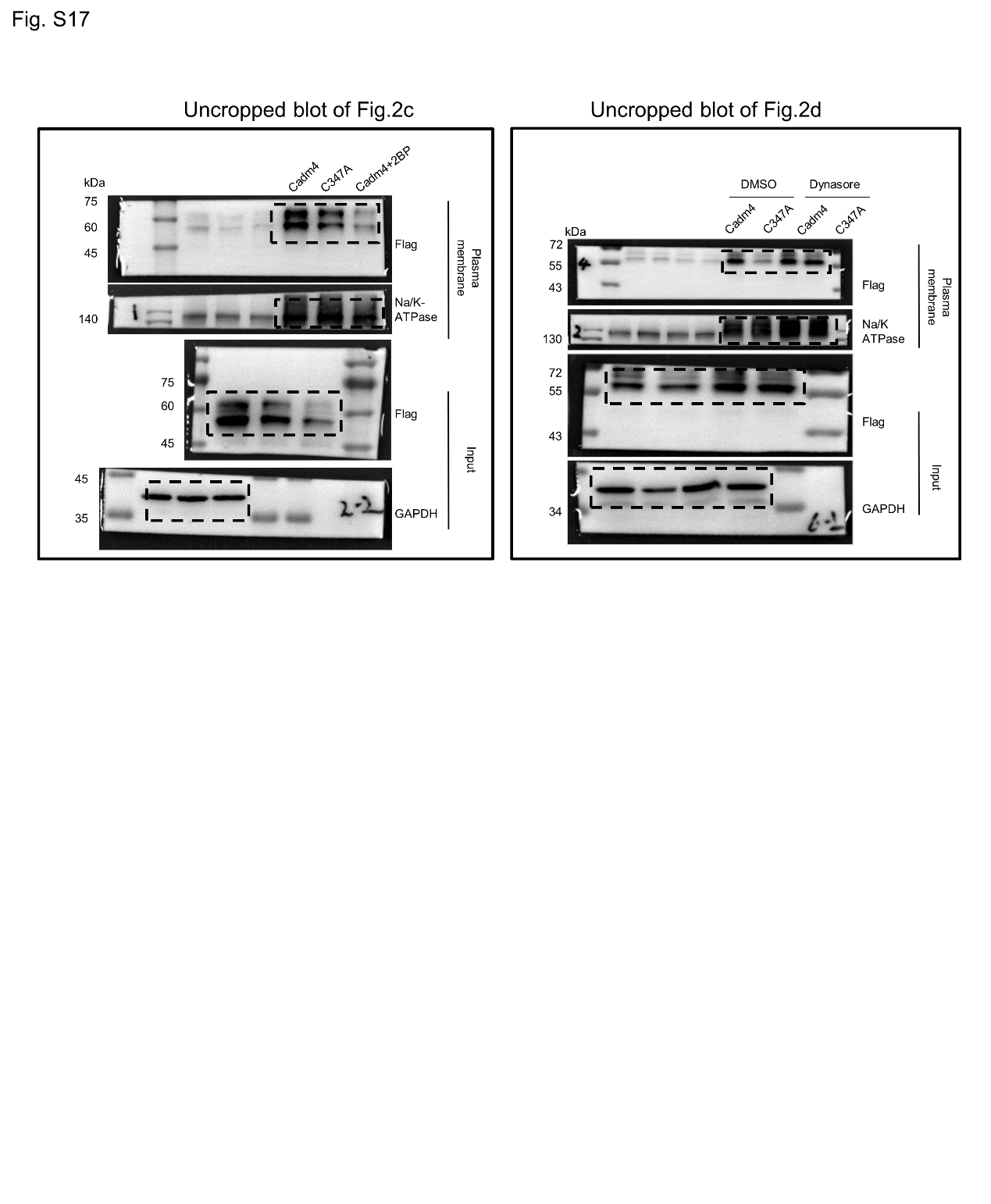
**

**Fig. S17. Uncropped blots for Fig. 2.**

**
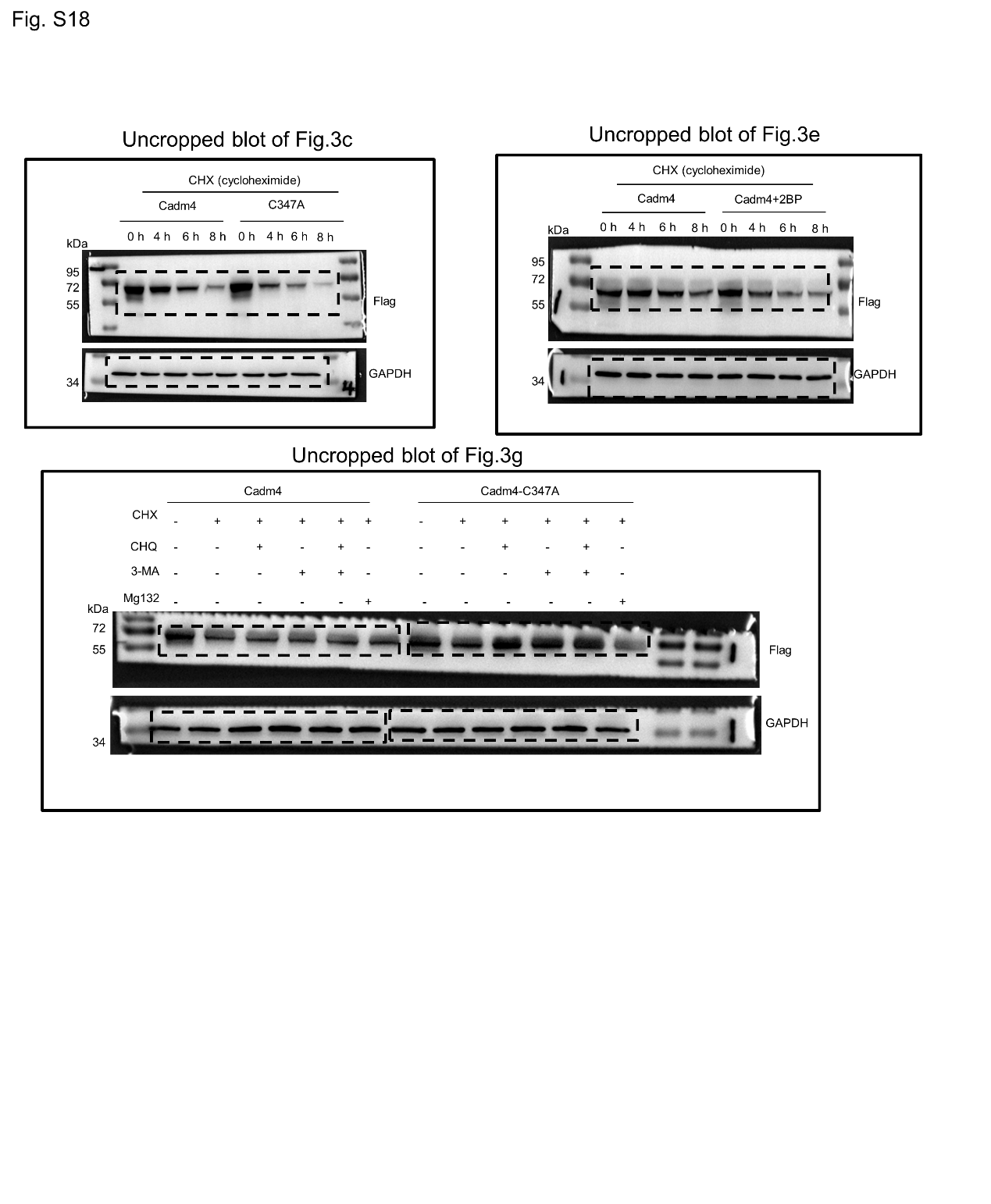
**

**Fig. S18. Uncropped blots for Fig. 3.**

**
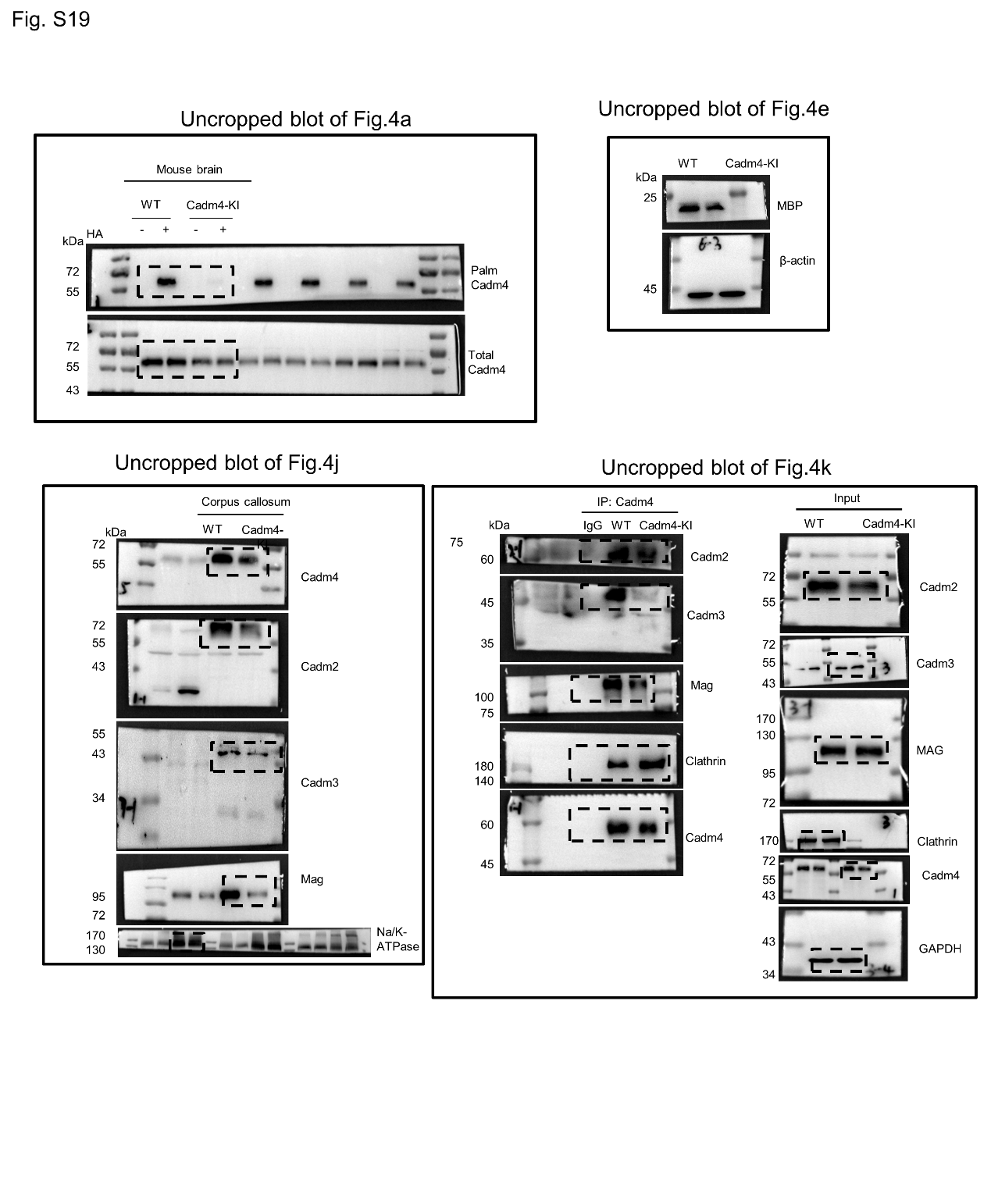
**

**Fig. S19. Uncropped blots for Fig. 4.**

**
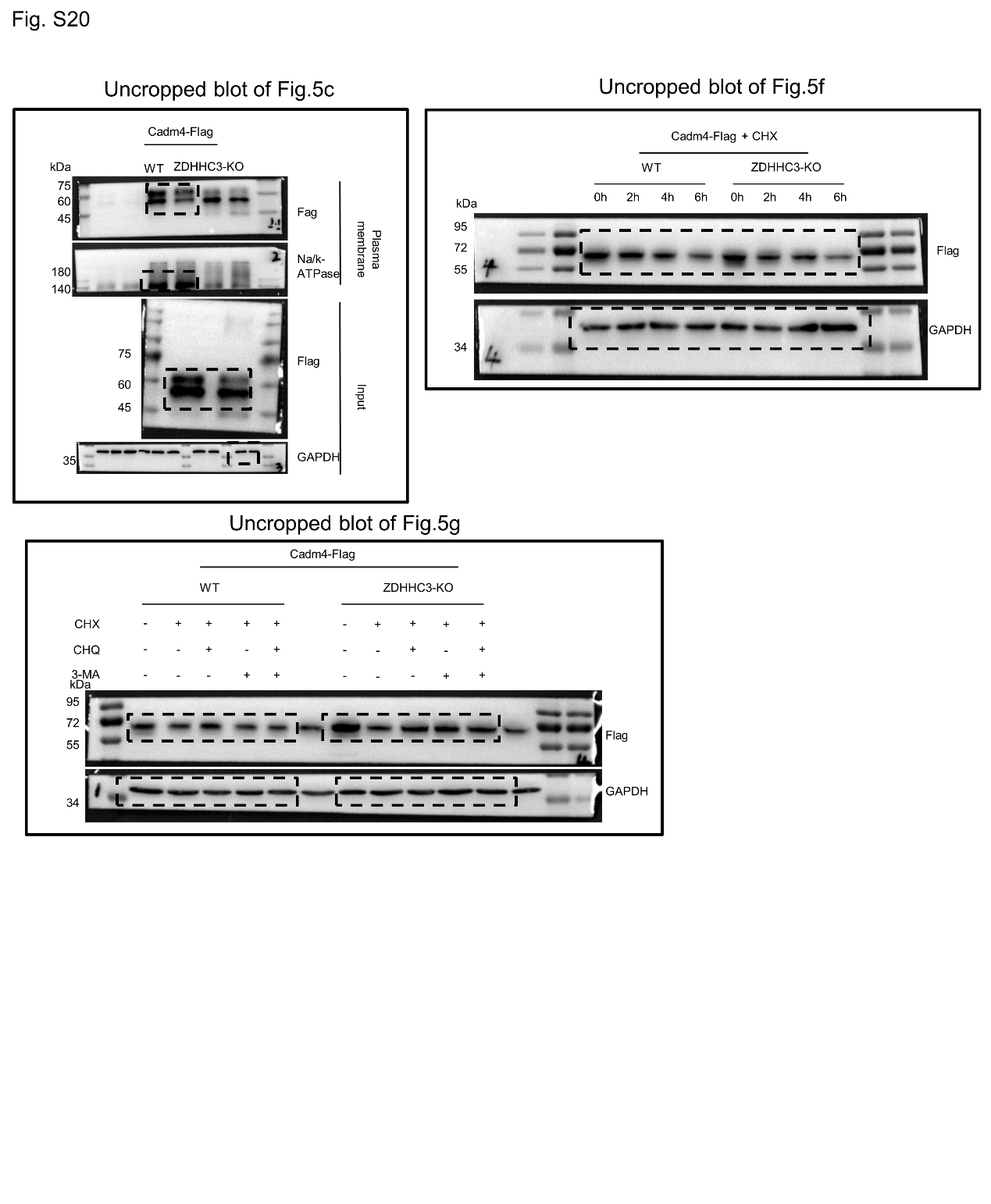
**

**Fig. S20. Uncropped blots for Fig. 5.**

**
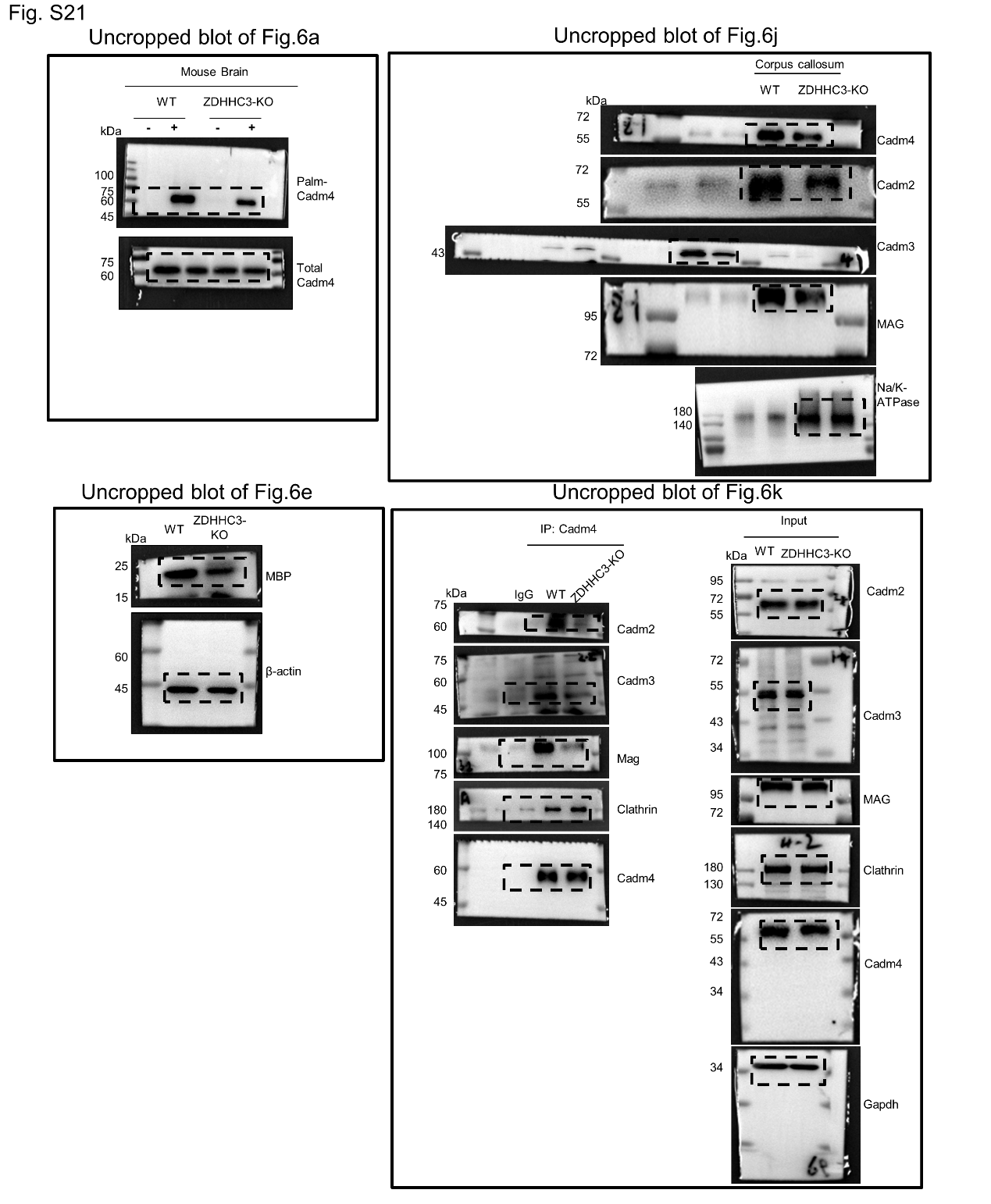
**

**Fig. S21. Uncropped blots for Fig. 6.**

**
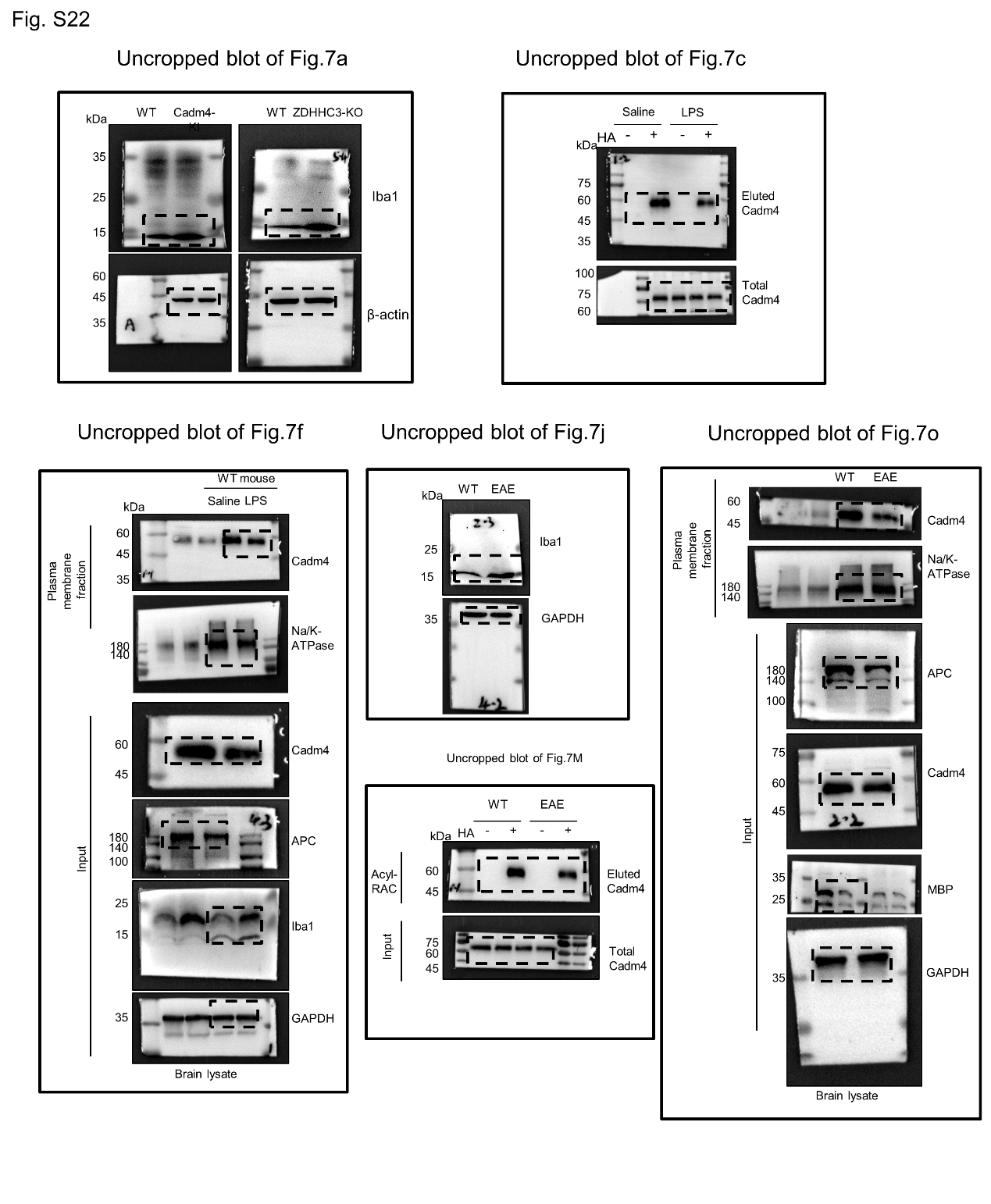
**

**Fig. S22. Uncropped blots for Fig. 7.**
